# Photocatalytic Treatment of Real Sugar Industry Wastewater Using Lignocellulosic Biomass-Derived Hydrochar/g-CN

**DOI:** 10.1021/acsomega.6c02485

**Published:** 2026-06-16

**Authors:** Bahriyenur Arabacı, Aslı Yüksel, Canan Varlıklı

**Affiliations:** † 52972 Izmir Institute of Technology, Department of Chemical Engineering, 35430 Urla, Izmir, Turkiye; ‡ Izmir Institute of Technology, Department of Photonics, 35430 Urla, Izmir, Turkiye

## Abstract

Hydrochar obtained from biomass via hydrothermal carbonization (HTC) was employed as a supporting material to enhance the photocatalytic activity of graphitic carbon nitride (g-CN). To the best of our knowledge, this is the first study combining sugar beet pulp-derived hydrochar with g-CN for photocatalytic treatment of real sugar industry wastewater, representing a circular economy approach where the solid waste of the sugar industry is valorized to treat its own liquid effluent. HTC process conditions were systematically optimized through Box-Behnken design with a second-order polynomial response surface model to examine the effects of temperature (200–260 °C), reaction time (60–120 min), and biomass amount (10–20 g). Operational parameters of pH (4–8), catalyst loading (0.2–1 g/L), and H_2_O_2_ (0–20 mM) were utilized during the optimization. The highest hydrochar yield (36.74%) was achieved at 200 °C for 60 min using 20g biomass. Under optimized photocatalytic conditions (0.81 g/L catalyst loading, pH 8, 20 mM H_2_O_2_), total organic carbon (TOC) removal efficiency reached 14.02%; the low removal rates are attributable to the exceptionally high initial TOC concentration (>7000 m/L), which far exceeds organic loads typically encountered in photocatalytic studies. Hydrochar incorporation improved the reusability and stability of the photocatalyst, enhancing resistance under complex wastewater matrix conditions. The results highlight the potential of biomass-derived hydrochar as a low-cost and sustainable strategy for improving photocatalytic treatment performance in industrial wastewater applications.

## Graphical abstract

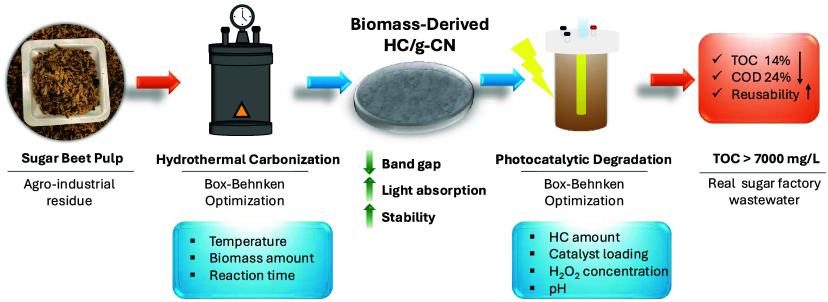

## 1 Introduction

Water contamination caused by organic molecules from industrial and agricultural effluents continues to be a major global concern, while freshwater systems are highly affected worldwide.^1,2^ Conventional treatment techniques, such as coagulation, adsorption, and biological degradation, can produce hazardous byproducts and are frequently insufficient for fully mineralizing these resistant chemicals, in addition to being costly and energy intensive.^3–5^ By producing reactive oxygen species in response to light, heterogeneous photocatalysis has become a viable, sustainable alternative that can break down a wide range of organic contaminants.^6–8^ The effectiveness of photocatalysis depends on the properties and amount of the catalyst, as well as light intensity.^9^ While increasing catalyst quantity boosts pollutant degradation, excess solid, heterogeneous catalyst particles can reduce efficiency due to light scattering effects that limit photon penetration into the reaction medium.^10^ Lowering the photocatalyst’s band gap enhances visible-light absorption, allowing more efficient utilization of solar energy.^9,11^ Nevertheless, the majority of photocatalytic research has been carried out under idealized conditions utilizing synthetic single-pollutant solutions, which do not accurately represent the complexity of actual wastewater matrices with coexisting pollutants, fluctuating pH, and competing organic matter. The development of photocatalysts that continue to function well under such practical circumstances is still a significant and unsolved challenge.

The integration of carbon-based materials with semiconductor photocatalysts such as TiO_2_ and ZnO enhances photocatalytic efficiency by improving surface adsorption, increasing active sites, and enabling better charge separation.^12^ However, traditional carbon sources are often nonrenewable or require hazardous, energy-intensive processes.^13^ This has led to research into more sustainable alternatives, such as carbon materials derived from biomass using hydrothermal carbonization (HTC). HTC transforms wet biomass into hydrochar (HC), a carbon-rich solid, and a liquid containing dissolved organics, with process parameters like temperature and duration influencing yield and quality. Studies have shown that higher HTC temperatures increase HC carbon content and energy value, making them suitable for fuel. The type of biomass used also affects the properties of the HC, such as aromaticity and functional groups. Optimization of HTC conditions can maximize HC yield and carbon content, demonstrating that both the severity of the process and the feedstock nature significantly impact the final product’s physicochemical properties.^14–20^ Due to its ability to process high-moisture feedstocks without predrying, HTC is suitable for agro-industrial residue like sugar beet pulp (SBP).^21–23^

Graphitic carbon nitride (g-CN) is a promising, sustainable, metal-free photocatalyst with good visible light activity and stability. It has been extensively investigated for various photocatalytic applications, and is suited for hydrogen evolution, selective organic oxidations, and pollutant degradation under visible light due to its heptazine-based conjugated structure, which has a bandgap of about 2.7 eV and band positions that straddle the redox potentials of water.^24,25^ For example, using oxidized g-CN, Wang et al. showed how sulfides may be selectively oxidized to sulfoxides. By increasing spin–orbit coupling and decreasing the singlet–triplet energy gap, integrated carbonyl groups improved singlet oxygen formation.^26^ Biochar-coupled g-CN nanocomposites have demonstrated efficacy in environmental treatment of a variety of harmful substances, such as antibiotics, dyes, heavy metals, and pharmaceuticals, by leveraging the enormous surface area and electron-transport properties of biochar to enhance charge separation.^27^ Additionally, under visible-light exposure, single-atom metal catalysts based on mesoporous g-CN degraded gemfibrozil by more than 90%.^28^ Through photocatalytic processes such as singlet oxygen generation, proton-coupled electron transfer, and photo charging, g-CN has also shown promise in chemical synthesis.^29^ However, it suffers from poor electrical conductivity and rapid charge recombination, which restricts its photocatalytic performance.^10^ Combining g-CN with biomass-derived carbon materials like HC and biochar has been proposed as an effective strategy to enhance interfacial charge transfer and pollutant adsorption. Although g-CN/biochar composites have been reported for model pollutant degradation,^30–34^ there is a research gap regarding HC-supported g-CN composites derived from HTC biomass for the treatment of real wastewater with high organic load and complex matrix composition, highlighting the need for further investigation.

On the other hand, the design of experiments (DoE) has emerged as an essential statistical methodology for optimization studies, offering a structured approach to understanding cause-and-effect relationships between multiple variables and process outputs simultaneously.^35–37^ However, selecting the appropriate DoE model is not straightforward, as different designs vary considerably in accuracy, resolution, and efficiency depending on the number of factors, their nature, and the extent of nonlinearity in the system. Among the variable designs, response surface methodology (RSM)-based designs, such as Box-Behnken design, are particularly well-suited for optimization purposes due to their capability of modeling quadratic responses and capturing curvature in the system behavior, while requiring a moderate number of experimental runs.^38,39^ Furthermore, DoE-based optimization not only identifies optimal operating conditions but also provides valuable insights into variable interactions and the relative significance of each factor, which would otherwise remain undetected with single-variable approaches.^35,38^ In this study, sustainable photocatalytic materials were developed by integrating SBP-derived HC with g-CN. The effects of HTC parameters (temperature, reaction time, and biomass amount) on HC production were systematically optimized using Box-Behnken experimental design. Subsequently, the influence of HC incorporation and operation conditions (pH, catalyst loading, and hydrogen peroxide concentration) on photocatalytic performance was evaluated. Unlike most studies that use synthetic dyes, this research evaluates photocatalytic performance using actual high-organic-load sugar beet wastewater under simulated irradiation.

## 2 Materials and Methods

### 2.1 Materials

Sugar beet pulp and sugar beet wastewater were obtained from Türkşeker Eskişehir Sugar Factory (Eskişehir, Türkiye). The SBP was ground using a knife mill to achieve a particle size range of 400–750 μm. The ground samples were then dried in an oven at 105 °C for 24 h to reduce their moisture content. All chemicals, including ethanol, urea, hydrogen peroxide (H_2_O_2_), sodium hydroxide (NaOH), sodium chlorite (NaClO_2_), acetic acid, toluene, acetone, and hydrochloric acid (HCl) were purchased from Merck and used as received without further purification.

### 2.2 Chemical Composition of Sugar Beet Pulp

Chemical composition including, the cellulose, hemicellulose, and lignin content of SBP, was determined based on the method proposed by Teramoto et al.^40^ Soxhlet extraction was applied for 6 h using an ethanol/toluene mixture (1:2 v/v) to remove fats and organic molecules. After extraction, 2.5 g of sugar beet particles were treated at 75 °C in 150 mL of diluted acetic acid with 2 g of NaClO_2_ for four cycles of 1 h each. The obtained particles were washed with acetone and distilled water, then dried in a vacuum oven at 105 °C for 24 h before weighing to determine holocellulose content.

To determine the α-cellulose content, 1 g of holocellulose was treated with 17.5% NaOH solution for 40 min, followed by the addition of 25 mL of distilled water. After 5 min, the mixture was filtered, and the solid fraction was further treated with 40 mL of 10% acetic acid solution, filtered again, and washed with 1 L of boiling distilled water. The sample was vacuum-dried at 105 °C for 48 h and weighed to calculate the α-cellulose content. Hemicellulose content was obtained by subtracting the α-cellulose content from holocellulose.

Lignin content was determined using acid hydrolysis. Briefly summarizing, 1 g of defatted biomass was treated with 15 mL 72% sulfuric acid and stirred at room temperature for 4 h. The mixture was then diluted with 560 mL of distilled water, stirred, and filtered. The solid residue was washed with cold and hot water, vacuum-dried at 105 °C for 24 h, and weighed to determine lignin content.

### 2.3 Analysis of Sugar Factory Wastewater

Characterization of wastewater generated from the sugar production was performed using inductively coupled plasma mass spectrometry (ICP-MS, Agilent 7500ce), and the TOC content of untreated wastewater was determined using a TOC analyzer (Shimadzu TOC-Vcph TNM-1/SSM-5000). COD value of untreated wastewater was measured using a COD Vario Tube Test (Lovibond, 0–15000 mg/L). In addition, gas chromatography–mass spectrometry (GC-MS, Agilent 6890 N/5973 N Network, USA) analysis was performed to identify major compounds present in untreated wastewater.

### 2.4 Hydrothermal Carbonization Experiments

The HTC of SBP was carried out in a batch stainless steel reactor (Parr 5500 Series, SS-316, USA) with a total volume of 300 mL, a maximum temperature of 350 °C, and a maximum pressure of 207 bar. The reactor was equipped with a temperature controller and heater (Parr, 4836) and connected to a cooling system (PolyScience, 9505). The determined amount of biomass was loaded into the reactor along with 100 mL of distilled water. Prior to heating, the reactor was purged 10 times with nitrogen (N_2_) to remove residual gases. A heating rate of 7 °C/min was applied to reach the target temperature, and the holding time at the target temperature was set. A mechanical stirrer was used to ensure uniform mixing of the reaction medium.

To optimize the reaction parameters affecting HC synthesis (biomass amount, temperature, and reaction time), a Box-Behnken design (3 factors, 3 levels, 6 center points) was created using Minitab software. The independent parameters were temperature (200, 230, and 260 °C), reaction time (60, 90, and 120 min), and biomass amount (10, 15, and 20 g). The temperature range was selected as it encompasses the statistically significant range for lignocellulosic biomass carbonization.^15^ Reaction time was limited to 60–120 min to avoid excessive degradation of solid yield at prolonged residence times.^41^ Biomass amount was constrained by the reactor capacity. A total of 18 experiments were conducted, including a replicated center point to assess experimental error and model accuracy.

At the end of each experiment, the heater was turned off, and the reactor was cooled down to room temperature at a cooling rate of 6 °C/min. The obtained solid–liquid mixture was separated by filtration using a Whatman grade 307 filter paper. The filtrate was collected and stored at 4 °C for further use.

The solid residue was washed with distilled water and purified by Soxhlet extraction using ethanol to remove residual organic and inorganic compounds. The obtained solid was then dried at 105 °C for 24 h, and the yield of HC was calculated by the following equation

1 $$\mathrm{Hydrochar}\;\mathrm{yield}\;\left(\mathrm{wt}\;\%\right)=\frac{\mathrm{Mass}\;\mathrm{of}\;\mathrm{hydrochar}}{\mathrm{Mass}\;\mathrm{of}\;\mathrm{initial}\;\mathrm{biomass}}\times 100$$

### 2.5 Synthesis and Characterization of HC/g-CN Photocatalysts

HC obtained under optimum HTC conditions (200 °C, 60 min, 20 g biomass) was used as the supporting material for photocatalyst preparation. The HC/g-CN composites were synthesized via thermal polymerization of urea in the presence of HC. Predetermined amounts of HC (0.05, 0.10, or 0.15 g) were mixed with 20 g of urea in an agate mortar to ensure homogeneous dispersion, corresponding to different HC loading ratios. The mixtures were then transferred into covered porcelain crucibles and calcinated in a muffle furnace at 550 °C for 3 h under ambient air, with a heating rate of 5 °C/min. After the thermal treatment, the samples were allowed to cool to room temperature, washed with distilled water to remove any residual impurities, and then dried at 105 °C for 24 h. Finally, the dried samples were ground in a mortar to obtain fine HC/g-CN powders, ready for subsequent characterization and photocatalytic experiments.

The physicochemical properties of HC and HC/g-CN were characterized using various analytical techniques. Ultimate analysis (C, H, N, and S) was conducted using an elemental analyzer (CHNS-932, Leco, USA). Surface chemical composition and electronic states were analyzed by X-ray photoelectron spectroscopy (XPS, Thermo Scientific K-α). Surface morphology was examined using scanning electron microscopy (SEM, 250 Fei Quanta 250 FEG USA), coupled with energy dispersive X-ray spectroscopy (EDX, ZEISS EVO 10) for elemental mapping. Functional groups were identified by Fourier transform infrared spectroscopy (FTIR, PerkinElmer Spectra Two, USA). was used to identify functional groups. Crystalline structures were analyzed using X-ray diffraction (XRD, Philips X’Pert Pro). Specific surface area was determined using the Brunauer–Emmett–Teller (BET) method using N_2_ adsorption–desorption isotherms at 77 K (Micromeritics Gemini V). Pore size distribution and total pore volume were calculated from the desorption data using the Barrett–Joyner–Halenda (BJH) method (Micromeritics 3Flex). The thermal stability and weight loss behaviors were evaluated by thermogravimetric analysis (TGA, Shimadzu, TGA-51) with changing temperature from 20 to 1000 °C at 10 °C/min in a 10 mL/min N_2_ flow atmosphere. Besides, TGA was used to obtain volatile matter (VM), fixed carbon (FC), and moisture compositions as well, while ash content was determined using a muffle furnace at 600 °C for 4 h, to conduct proximate analysis. Optical properties, including light absorption and emission characteristics, were investigated by photoluminescence spectroscopy (PL, PerkinElmer, L 55) and UV–vis absorption spectroscopy (PerkinElmer, Lambda 25).

### 2.6 Photocatalytic Degradation of Sugar Factory Wastewater

The photocatalytic degradation of sugar factory wastewater was investigated by the synthesized HC/g-CN as photocatalyst under simulated solar radiation provided by a Philips-Master PL-L (24 W, 1800 luminous flux). The irradiance was measured using an ABET RR-1002 reference cell and determined to be 10–15 mW/cm^2^. Experiments were conducted in a cylindrical glass reactor (*V* = 1 L), equipped with a magnetic stirrer and connected to a refrigerated circulator (CLS Scientific, CLRC-08C) to maintain constant temperature during reaction. The degradation efficiency was evaluated based on TOC and COD analyses.

For each experiment, 300 mL of real wastewater was used as the reaction medium, and the pH was adjusted using 0.1 M HCl or 1 M NaOH solutions. The desired amount of HC/g-CN and H_2_O_2_ was then added. Prior to light irradiation, the suspension was magnetically stirred in the dark for 30 min to establish adsorption–desorption equilibrium. Subsequently, the lamp was switched on to initiate the photocatalytic reaction, which was maintained for 120 min under continuous stirring at room temperature. At predetermined intervals, samples were collected, filtered through 0.45 μm membrane filters, and analyzed for TOC and COD values to determine degradation efficiency. To statistically evaluate the impact of process parameters, a Box-Behnken model was applied using Minitab software. The independent variables were pH (4 and 8), catalyst loading (0.2 and 1.0 g/L), H_2_O_2_ concentration (0 and 20 mM), and HC loading amount (0.05 and 0.15 g). The operational parameter ranges were selected based on published literature on photocatalytic treatment of sugar beet wastewater to encompass conditions previously reported to influence hydroxyl radical generation, light distribution, and oxidative efficiency in similar systems.^42^ The center point was replicated six times to estimate pure error and evaluate model adequacy. The model was used to assess individual and interaction effects of the variables and to determine the optimal conditions for photocatalytic degradation. The degradation kinetics were evaluated using a pseudohomogeneous first-order kinetic model. The rate constants (*k*) were determined from the linear regression of ln­(*C*_0_/*C*_t_) versus reaction time. Reusability tests were conducted over three consecutive cycles under optimized conditions. After each cycle, the catalyst was recovered by filtration, washed with distilled water, dried, and reused in the subsequent experiment.

## 3 Results and Discussion

### 3.1 Characterization of Sugar Beet Pulp and Sugar Factory Wastewater

The higher heating value (HHV) of raw sugar beet pulp, calculated via Dulong’s formula,^43^ was determined to be 16.05 MJ/kg. The characterization results are summarized in Table 1 and are consistent with values reported in the literature.^44,45^ GC-MS analysis (Tables S1 and S2) indicated a high content of oxygenated organic compounds, predominantly 1,2-propanediol (34.83%) and acetic acid (19.88%), trace amounts of alcohols, carboxylic acids, and ketones. Furthermore, elemental analysis of the sugar beet wastewater showed high levels of Ca (338.4 mg/L), K (324.0 mg/L), and Na (62.63 mg/L), as well as a substantial total nitrogen content (312.0 mg/L). Trace metals such as Fe, Mn, Zn, and Cu, as well as toxic elements such as Pb and As, were also present. The observed chemical composition is consistent with typical sugar-processing effluents reported in the literature, in which high concentrations of light metals are attributed to natural plant uptake and the routine use of alkaline reagents (e.g., Ca­(OH)_2_ and NaOH) during processing. Moreover, the significant Ni load and the predominance of oxygenated organic compounds, particularly acetic acid, are characteristic of sugar industry wastewater, reflecting the hydrolysis and acidification of readily biodegradable carbohydrates into volatile fatty acids.^46–48^

**Table 1:** Sugar Beet Pulp Characterization Results

Chemical Compositions (%)	
Cellulose	22.61
Hemicellulose	31.11
Lignin	27.70
Extractives	18.58
Proximate Analysis (%)	
Moisture^a^	4.17
Ash^b^	2.67
Volatile Matters (VM)^a^	97.33
Fixed Carbon (FC)^a^ ^,^ ^c^	0
Ultimate Analysis (%)	
C	41.87
H	5.93
N	1.50
S	0
O	48.3
HHV (MJ/kg)	**16.05**

a Determined by TGA.

b Determined using a muffle furnace at 600 °C for 4 h in static air.

c FC = 100% − VM% − Ash%.

### 3.2 Optimization of Hydrothermal Carbonization Conditions

The optimization experiments for maximizing HC production via HTC were conducted using a Box-Behnken design generated in Minitab. In this design, six replicate experiments were performed at the center point to ensure model reliability. The selected process variables were biomass amount, reaction temperature, and reaction time. The uncoded levels of these factors and the corresponding experimental results are presented in Table 2.

**Table 2:** Uncoded Values of Factors and the Corresponding Experimental Results

Number of Experiments	Amount of SBP (g)	Reaction Temperature (^o^C)	Retention Time (min)	Hydrochar Yield (%)
1	15	200	120	34.04
2	15	200	60	32.58
3	10	200	90	32.28
4	10	230	60	26.12
5	10	260	90	19.18
6	20	230	60	33.76
7	20	230	120	32.56
8	15	260	60	23.21
9	10	230	120	30.19
10	15	260	120	23.62
11	20	260	90	21.98
12	20	200	90	34.94
13	15	230	90	29.84
14	15	230	90	28.59
15	15	230	90	28.91
16	15	230	90	30.36
17	15	230	90	27.68
18	15	230	90	27.59

Table S3 shows that the overall model was statistically significant (*F* = 21.01, *p* < 0.001), showing a strong relationship between the chosen variables and HC yield. Among the linear terms, temperature was the most influential factor (*p* < 0.001), followed by biomass amount (*p* = 0.003), whereas reaction time was not significant (*p* = 0.239). An insignificant time effect can be explained by the fact that at shorter reaction times, a considerable fraction of the biomass is solubilized, with hydrochar formation declining, then increasing over time, and balancing off to a maximum.^49^ In this way, Li et al. and Baoteng et al. reported that the effect of reaction time is not remarkable on HC yield.^50,51^ Furthermore, the quadratic terms, particularly for temperature (*p* = 0.013) and time (*p* = 0.040), were significant, suggesting nonlinear relationships for these parameters. However, the quadratic term for biomass amount was not significant (*p* = 0.668), implying a mostly linear effect. No substantial two-way interactions were detected at α = 0.05. However, the interaction between biomass quantity and time was statistically significant at the 90% confidence level (*p* = 0.081), confirming that these parameters exhibit a coupled effect on HC yield. The absence of significant interactions for the other parameter combinations implies that under tested moderate HTC conditions, individual parameter effects are dominant, and interactions may emerge under more extreme process conditions. Six center replicates were included in the design to estimate the pure error mean square of 1.257 (df = 5), and the nonsignificant lack-of-fit test result (*F* = 2.02, *p* = 0.229) confirmed the model’s robust fit to the experimental data. Also, the determination coefficient (*R* ^2^ = 0.9594) confirmed that the predicted values closely matched the actual HC yields. The Pareto chart (Figure S1a) illustrates the standardized effects of each factor and their interactions on HC yield, with a reference line at 2.31 revealing statistical significance. Temperature (B) exceeded the reference line by the largest margin, confirming the dominant role. Biomass amount (A) and the quadratic terms for temperature (BB) and time (CC) also surpassed the significance threshold, revealing nonlinear relationships for these parameters. In contrast, reaction time (C) and all two-way interaction terms (AB, AC, BC) remained below the reference line, indicating their negligible contribution to HC yield variation under the tested conditions. The normal probability plot of residuals (Figure S1b) showed that the residuals followed a straight line, with a minor deviation at the lower tail, confirming that the model assumptions of normality and homoscedasticity were satisfied and that no significant outliers were present in the data set.

Figure 1 depicts response surface plots showing the interactive effects of temperature, biomass quantity, and reaction duration on HC yield. When the reaction time was fixed at 90 min, HC yield increased with biomass amount, while lower temperatures enhanced the yield (Figure 1a). Similarly, at a fixed biomass amount of 15 g, yield was higher at lower temperatures, whereas reaction time had a limited effect (Figure 1c). However, HC yield decreased noticeably as the HTC temperature and reaction time increased, which may be due to extensive breakdown of biomass structures or further degradation of the formed solid residues, as noted in previous studies.^20,52^ When the temperature was held constant at 230 °C, the HC yield showed a nonlinear trend with respect to reaction time, initially decreasing and then increasing again, while biomass amount showed a linear positive effect (Figure 1b). Overall, the plots confirm that biomass amount consistently contributes positively to HC yield, while temperature exerts a more complex influence depending on its interaction with other variables.

**Figure 1:**
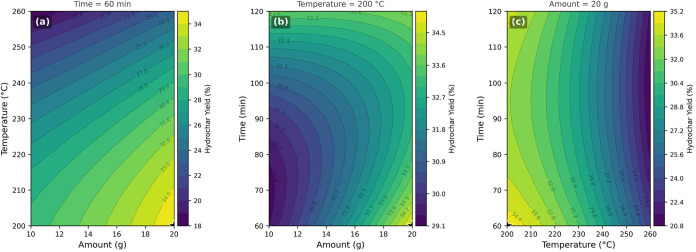
Response surface plots showing the interactive effect of (a) amount and temperature, (b) temperature and time, and (c) amount and time on hydrochar yield (%) (other variables fixed at optimum).

Previous studies have reported that mild HTC conditions favor higher HC mass recovery. Increasing temperature and prolonging reaction time intensify hydrolysis, dehydration, and decarboxylation reactions, promoting the conversion of organic matter into soluble intermediates and gaseous products and thereby reducing solid yield. In contrast, lower temperatures and shorter reaction durations limit excessive devolatilization and structural fragmentation, preserving a larger fraction of the original carbon within the solid matrix. These considerations support the observed trend that HC yield decreases under more severe HTC conditions.^15,53–56^

Based on numerical optimization, the highest predicted HC yield of 36.74% was achieved at 20 g of biomass, 200 °C, and 60 min reaction time, as demonstrated by the star marker in Figure 1. Experimental validation under these optimum conditions yielded an HC yield of 37.62%, corresponding to a relative error of 2.40%, confirming the predictive reliability of the developed model. A new HC batch was synthesized under these optimum conditions and used as the support material for the synthesis of HC/g-CN composites in subsequent experiments.

### 3.3 Characteristics of Hydrochar Samples

Although 18 HC samples were prepared according to the Box-Behnken design, 6 were replicated at the center point. Since these center-point experiments were conducted under identical conditions and yielded highly similar results, only the 13 distinct experimental conditions were presented in this section.

#### 3.3.1 Elemental Analysis of Synthesized Hydrochar

Elemental and proximate analyses were performed to assess the degree of carbonization and chemical transformation of the raw biomass upon hydrothermal treatment, with the higher heating value as an indicator in terms of energy conversion. The results of elemental composition and proximate analysis were summarized in Table S4. The carbon content increased from 41.87% in the raw biomass to 68.02% in HC10 (260 °C, 15 g, 120 min), while the oxygen content dropped notably. This shows that carbonization involved reactions such as dehydration and decarboxylation, as reported in earlier studies.^17,57^ Also, these changes demonstrate that the substance became more carbon-rich and thermally stable as reaction conditions became harsher. At the same time, the HHV increased from 16.05 MJ/kg to as much as 26.35 MJ/kg for HC11 (260 °C, 20 g, 90 min).

#### 3.3.2 Sem Images of Synthesized Hydrochar

SEM was employed to examine the morphological evaluation of the samples by providing outputs from structural transformation during the applied process. Figure 2 shows the morphological changes of sugar beet pulp after HTC under various conditions. The raw biomass exhibited a compact and fibrous structure. The HC obtained at 200 °C showed the onset of thermal decomposition, as indicated by slight surface degradation, visible shrinkage, and partial collapse of fiber walls. When the temperature increased to 230 °C, the surface became more disrupted and rougher with the appearance of irregular pores and openings. Compared to milder conditions, the HC surface became more irregular and degraded under more severe carbonization. At 260 °C, the original fibrous morphology was almost destroyed and replaced by a sponge-like architecture.^58^ Furthermore, comparison of HC5 and HC11 suggests that the lower biomass amounts may lead to more extensive degradation, likely due to more uniform and accelerated heat transfer in the reaction medium.

**Figure 2:**
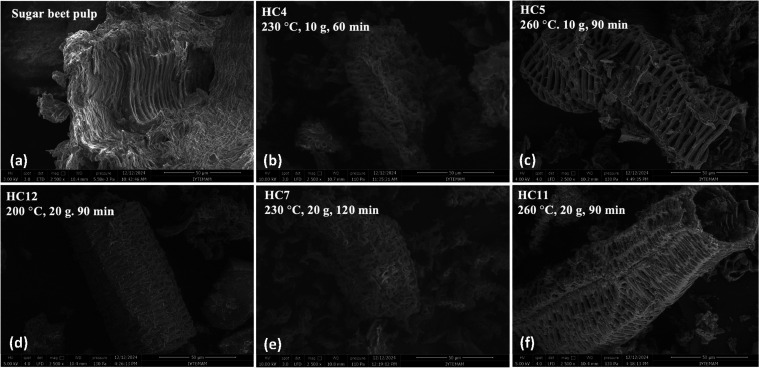
SEM images of (a) the sugar beet pulp and (b–f) HC samples under varying conditions.

#### 3.3.3 Functional Groups of Synthesized Hydrochar

FTIR was used to identify surface functional groups in the samples to provide molecular-level evidence of the chemical transformations. Figure 3 presents the representative FTIR spectra of selected HC samples (Biomass, HC2, HC4, and HC10), illustrating the key structural transformations induced by increasing temperature. The complete data set for all HC samples is provided in Figure S2. A broad band was observed around 3300 cm^–1^, corresponding to −OH stretching vibrations from the hydroxyl group. This peak weakened as the temperature increased because oxygen was removed during the dehydration reaction.^59^ Similarly, the aliphatic C–H stretching peak at 2923 cm^–1^ decreased in intensity, suggesting increased carbonization. The 1600–1500 cm^–1^ region exhibited peaks associated with aromatic C=C stretching, which became more intense with increasing temperature. The spectra also showed a distinct peak at 1700 cm^–1^, assigned to carbonyl (C=O) stretching vibrations, which decreased as the temperature increased.^60^ Additional structural modifications are observed in the 1200–950 cm^–1^ region, where the weakening of peaks associated with C–O and C–O–C stretching vibrations indicates the formation of a more condensed carbon framework. Overall, increasing HTC temperature leads to the loss of hydroxyl, aliphatic, and carbonyl groups, while increasing aromaticity, deoxygenation, and graphitization.^58^

**Figure 3:**
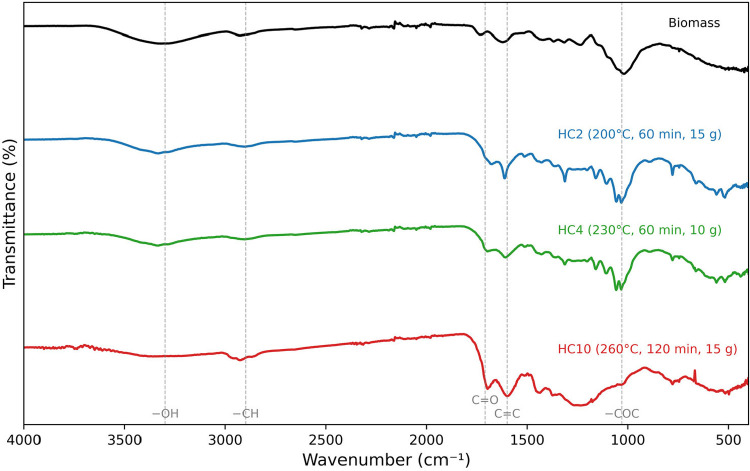
FTIR spectra of biomass and representative HC samples synthesized under different HTC conditions: HC2 (200 °C, 60 min, 15 g), HC4 (230 °C, 60 min, 10 g), and HC10 (260 °C, 120 min, 15 g).

#### 3.3.4 Crystallinity of Synthesized Hydrochar

XRD analysis was performed to evaluate the crystallinity and structural ordering of the samples, representing the degree of graphitization and the transformation. Figure 4 presents representative XRD patterns of selected HC samples (Biomass, HC2, HC4, and HC10); the complete data set for all HC samples is provided in Figure S3. The diffraction peaks observed at 15.65°, 22.45°, 35.15°, 38.2°, and 44.75° correspond to the typical crystalline features of hydrochars, which are attributed to (012), (104), (040, (113), and (202) planes, respectively.^61^ The raw biomass exhibited a broad diffraction peak at around 22°, indicating its largely amorphous nature. After HTC, noticeable changes occurred in both peak intensity and shape. At 200°, the peaks became sharper and more distinct with increasing residence time. As the HTC temperature was raised to 230° and 260°, diffraction peaks around 26° began to emerge in some samples, confirming the development of more graphitic-like structures. The increase in crystallinity can be explained by the partial removal of amorphous components such as hemicellulose and lignin, which caused more ordered carbon structures.^58^

**Figure 4:**
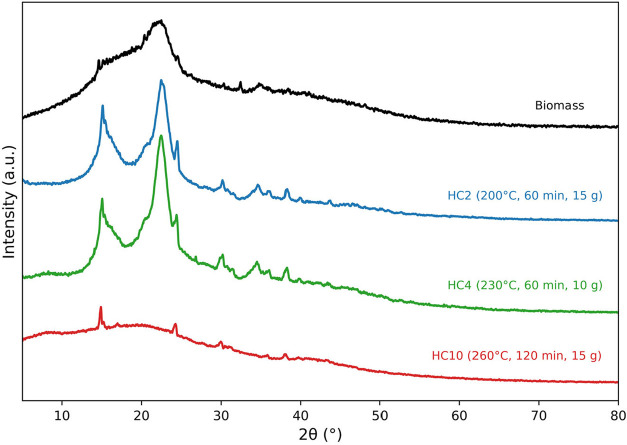
XRD pattern of biomass and representative HC samples synthesized under different HTC conditions: HC2 (200 °C, 60 min, 15 g), HC4 (230 °C, 60 min, 10 g), and HC10 (260 °C, 120 min, 15 g).

#### 3.3.5 Surface Area of Synthesized Hydrochar

BET surface area analysis was conducted to quantify the porosity of the samples, as a higher surface area provides more accessible active sites for pollutant adsorption and photocatalytic reactions. Figure 5 presents the BET surface area (m^2^/g) results of HC samples synthesized at different conditions. The BET surface area of the sugar beet pulp was only 0.5 m^2^/g, which increased after HTC. The highest BET surface area was observed for HC12 (20 g, 200 °C, 90 min), followed by HC13 (15 g, 230 °C, 90 min), revealing that moderate temperature and reaction time result in the development of porous structures. It could be explained by the hemicellulose breakdown, cellulose depolymerization, and partial lignin degradation. On the other hand, at higher temperatures and longer durations, a decrease in surface area was observed due to the acceleration of structure decomposition. Similar trends have been reported in the literature, where HTC significantly increases the porosity and BET surface area of biomass compared to its raw form.^17^ For example, the BET surface area of oil palm shells rises from 0.36 to 12.6 m^2^/g after HTC at 260 °C, while date seed-derived HC reaches 60.75 m^2^/g after treatment at 200 °C for 120 min.^62–64^

**Figure 5:**
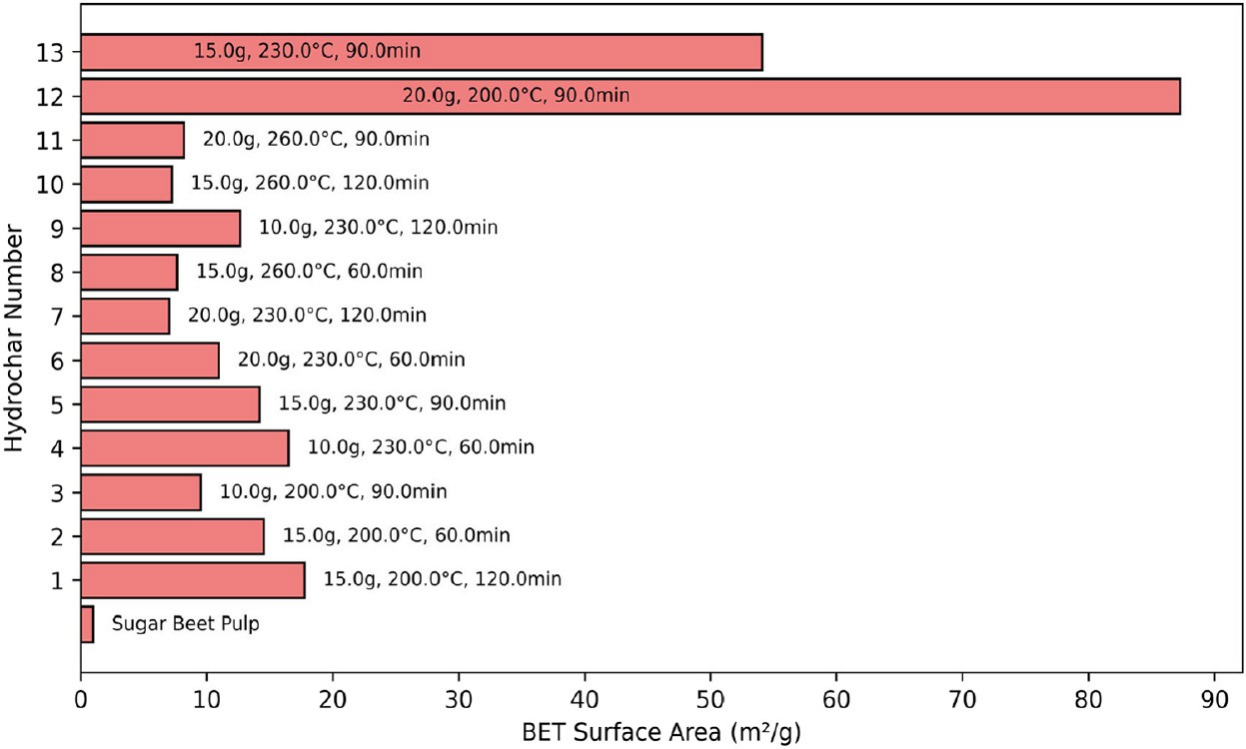
BET surface area of HC samples at different experimental conditions.

#### 3.3.6 Thermal Stability of Synthesized Hydrochar

The thermal stability and degradation behavior of the samples were assessed by TGA analysis. Derivative thermogravimetric (DTG) and thermogravimetric (TG) curves of the biomass and HC (Figure S4a–c) show that the thermal degradation behavior changed with temperature, time, and biomass amount. According to the DTG curves, samples synthesized at 200 °C exhibited earlier decomposition peaks, while those obtained at 260 °C showed sharper peaks shifted to higher temperatures. Moreover, TG curves showed that weight loss occurred at higher temperatures with increasing synthesis temperature, indicating a higher degree of carbonization and improved thermal stability (Figure S4e,f). Similar thermal behavior was also reported in previous studies, where HC samples exhibited a three-step degradation: an initial weight loss due to moisture evaporation below 110 °C, followed by the breakdown of hemicellulose and cellulose between 250 and 400 °C, and finally a slower degradation phase attributed to lignin and inorganic matter from 400 °C to around 700 °C.^65,66^

### 3.4 Characterization of Synthesized HC/g-CN Photocatalysts

#### 3.4.1 Morphological and Textural Analysis of HC/g-CN Photocatalysts

Figure 6 shows SEM images of g-CN, HC, and HC/g-CN composites with different HC loadings. The surface morphology of g-CN exhibited a dense and irregularly stacked structure with blocky agglomerates in the micrometer size range (Figure 6a). It should be noted that g-CN is not intrinsically porous as a graphitic material; the observed porosity and aggregate morphology are attributable to the use of urea as the synthesis precursor. During thermal decomposition, urea releases NH_3_ and H_2_O gases that act as in situ soft templates, generating a porous and less condensed structure.^67^ In contrast, the HC sample presented a more compact and rougher surface with larger, nonuniform particles (Figure 6b). After HC incorporation, the characteristic layered structure of g-CN remained observable. Notably, the 0.10 HC/g-CN composite exhibited a more porous surface morphology, whereas further increase in HC content led to denser particle aggregation and reduced surface uniformity (Figure 6c–e).^30^

**Figure 6:**
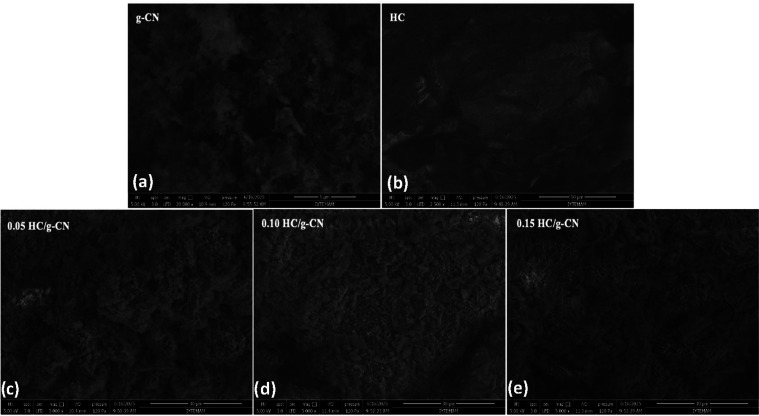
SEM images of (a) g-CN, (b) HC, and (c–e) HC/g-CN with different HC loading.

The XRD patterns of HC, g-CN, and HC/g-CN composites with different HC loadings are presented in Figure 7. For pure g-CN, two characteristic diffraction peaks were observed at around 12.9° and 27.5°, which corresponded to the (100) and (002) crystal planes, respectively.^68^ The XRD pattern of pure HC exhibited several sharper diffraction peaks, particularly in between the 10 and 30° 2θ range, reflecting its partially crystalline character. In the composites, these HC-associated features become less distinguishable, likely due to the dominant and broad g-CN diffraction pattern and relatively low HC loading, through the progressive broadening of the (002) plane with increasing HC content, which is consistent with the incorporation of the amorphous HC phase into the g-CN structure. The peak at 12.9° was attributed to the in-plane structural motif of tri-s-triazine units, while the strong peak at 27.5° was related to the interlayer stacking of the conjugated aromatic system. Upon the addition of HC to g-CN, the intensity of the (002) peak gradually decreased, due to the increased degree of amorphization of g-CN, as also reported in similar studies.^10,34^ Furthermore, the slight shift of the (002) peak toward lower 2θ values showed an expansion of the interlayer spacing in g-CN, which is beneficial for improving visible light absorption due to looser stacking.^69^ In addition, the small shift observed in the (100) peak suggested minor changes in in-plane structural arrangement.

**Figure 7:**
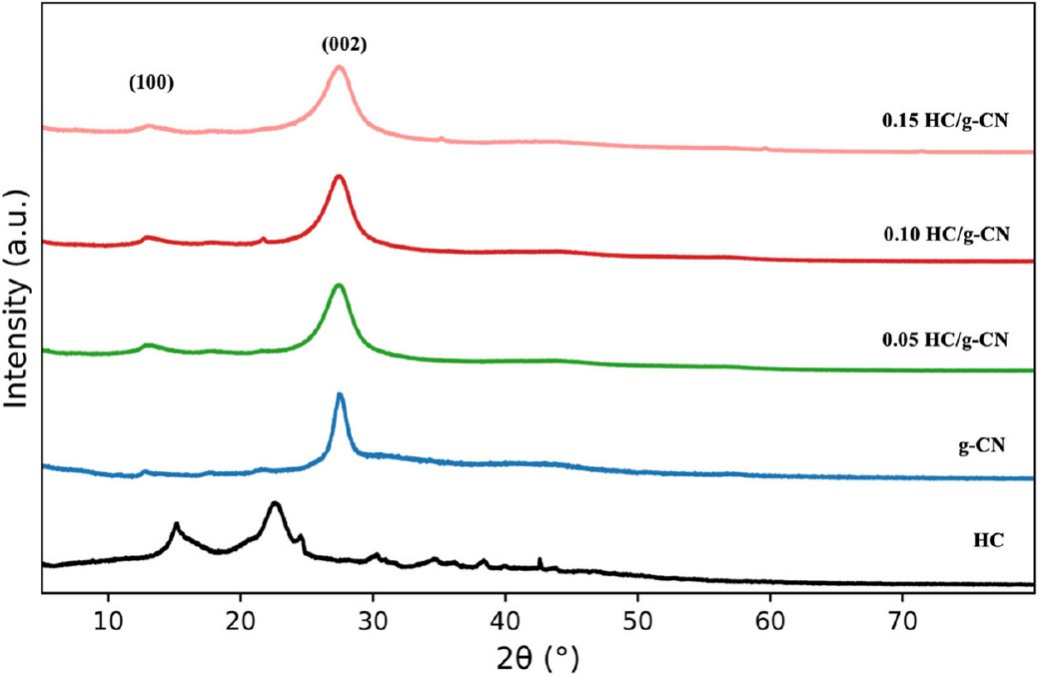
XRD Spectra of HC, g-CN, and HC/g-CN with different HC loading.

C/N molar ratios, BET surface areas, pore volumes, and radius of the pure HC, g-CN, and synthesized photocatalyst with different ratios (0.05, 0.10, and 0.15) are listed in Table 3. The highest C/N molar ratio was observed for HC, where nitrogen was below the detection limit, consistent with the carbon-rich nature of HC. The addition of HC increased the C/N ratio of g-CN (0.514) progressively to 0.665 composites, confirming the incorporation of the HC into the g-CN matrix with increasing HC content. Conversely, naturally pure g-CN exhibited the highest BET surface area at 119.75 m^2^/g. However, after HC addition, decrements were reported. However, while 0.05 and 0.15 were close at 71.92 m^2^/g and 69.61 m^2^/g, respectively, the composition with 0.10 HC/g-CN increased the BET surface area from 48.54 m^2^/g (for pure HC) to 88.54 m^2^/g by ensuring the least reduction compared to pure g-CN at the same time. Similarly, pore volume and average pore radius showed the same trend as BET surface area for the composites, reaching the highest values with 0.10 HC/g-CN, at 0.55 cm^3^/g and 28.23 nm, respectively. A larger pore size typically increases the accessibility of the active site, thereby improving photocatalytic efficiency. As the HC content increased from 0.10 to 0.15, the BET surface area, pore volume, and pore radius decreased, consistent with previous studies. This decline could mainly be attributed to the increasing dominance of HC within the composite structure at higher loadings, where excessive HC and the g-CN matrix blocked available pores. Additionally, potential CO_2_ release from HC during thermal treatment may cause a reduction by inducing pore collapse or structural densification.^34,70,71^ It is worth noting that the high BET surface area of pure g-CN (119.75 m^2^/g) is attributable to the use of urea as precursor; other precursors such as melamine and Dicyandiamide yield bulk g-CN with lower surface areas (10.59 and 6.05 m^2^/g, respectively).^67^

**Table 3:** Elemental Analysis, BET Surface Area, Pore Volume, and Radius of HC, g-CN, and Their Composites

Samples	C/N molar ratio^a^	BET (m^2^/g)	*V*_total_ (cm^3^/g)^b^	Ave-pore radius (nm)^b^
HC	-	48.5457	0.154550	12.4102
g-CN	0.514	119.7490	0.451432	26.9107
0.05 HC/g-CN	0.543	71.9149	0.415667	25.8550
0.10 HC/g-CN	0.581	88.5428	0.551671	28.2250
0.15 HC/g-CN	0.665	69.6115	0.362317	21.9006

a Calculated based on CHNS results.

b Pore volume, and pore diameter assigned to the specific surface area obtained via the multipoint BJH method.

The nitrogen adsorption–desorption isotherms and pore size distribution curves of HC, g-CN, and HC/g-CN composites with varying HC loading are shown in Figure 8. All samples exhibited type IV isotherms with H3-type hysteresis loops, characteristics of mesoporous structures with slit-like pores, typically formed by the agglomeration of plate-like particles (Figure 8a). The presence of this hysteresis behavior indicated that the addition of HC enhanced the growth of pore structure rather than changing the mesoporous character of g-CN. Furthermore, based on BJH pore size distribution curves, the presence of a narrow pore size distribution centered around 2–4 nm suggested that the samples predominantly consist of a mesoporous structure (Figure 8b). Such mesostructures provide a readily accessible pore-wall system and contribute to enhanced photocatalytic performance due to the confined space effect.^72^

**Figure 8:**
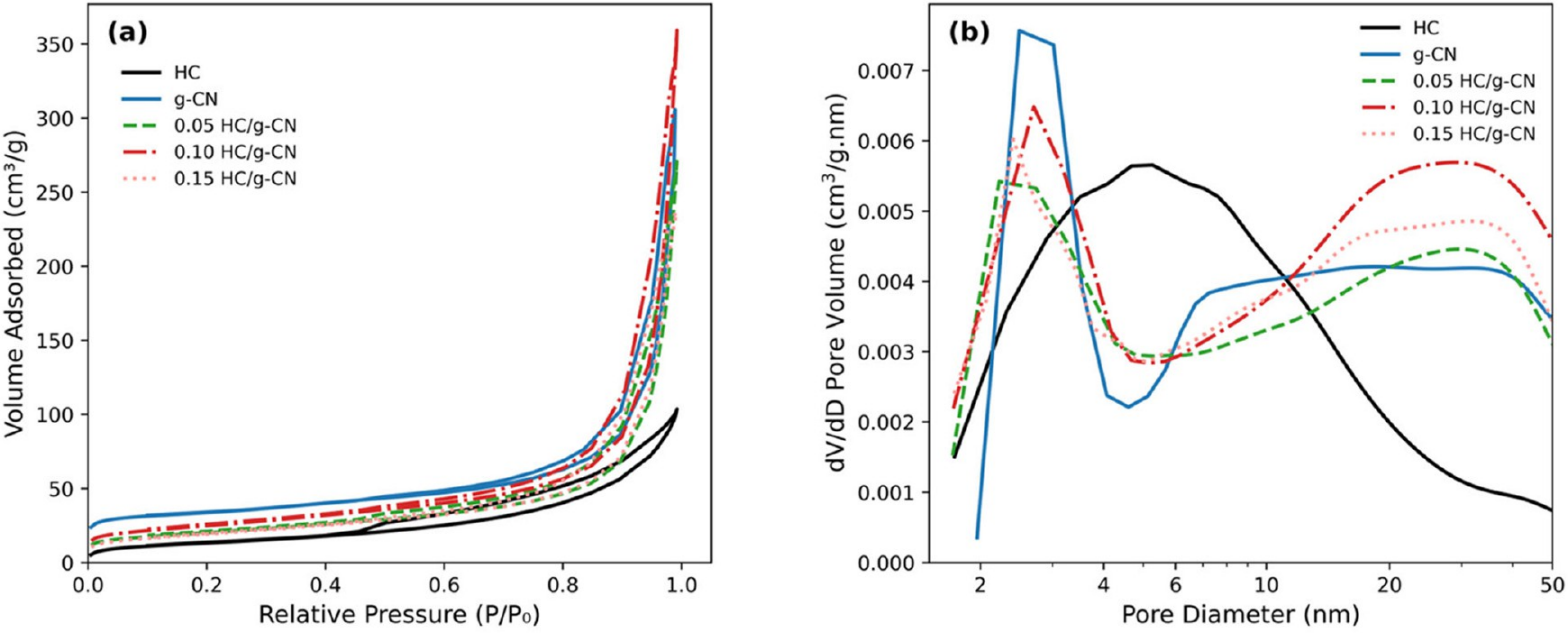
(a) Adsorption and desorption isotherms and (b) pore size distributions of HC, g-CN, and HC/g-CN with different HC loading.

#### 3.4.2 Chemical Analysis of HC/g-CN Photocatalysts

The functional groups and bonding of the synthesized samples were examined using FTIR spectroscopy, and the results are shown in Figure 9. The raw HC showed broad absorption bands between 3000 and 3700 cm^–1^ due to −OH and C–H stretching, and at 1078 cm^–1^ due to C–O–C, typical of lignocellulosic structures. Peaks in 1740 and 1600 cm^–1^ corresponded to C=O and C=C bonds from lignin.^65^ For the g-CN sample, a broad adsorption band in the 3000–3500 cm^–1^ range corresponded to N–H stretching vibrations, related to residual amine groups.^73^ The intense absorption between 1200 and 1650 cm^–1^ was attributed to C–N and C=N stretching in the aromatic heterocyclic framework of g-CN. A band at 810 cm^–1^ corresponded to the vibration of tri-s-triazine rings, which is a distinctive feature of the g-CN.^74^ Compared to pure g-CN, the HC/g-CN composites exhibited a broader absorption band in the 3000–3500 cm^–1^ range, which can be attributed to the stretching vibrations of N–H and O–H groups. Importantly, the composites displayed the characteristic absorption peaks of both HC and g-CN, with no additional peaks observed, which confirmed that no new functional groups were formed during synthesis.^10^

**Figure 9:**
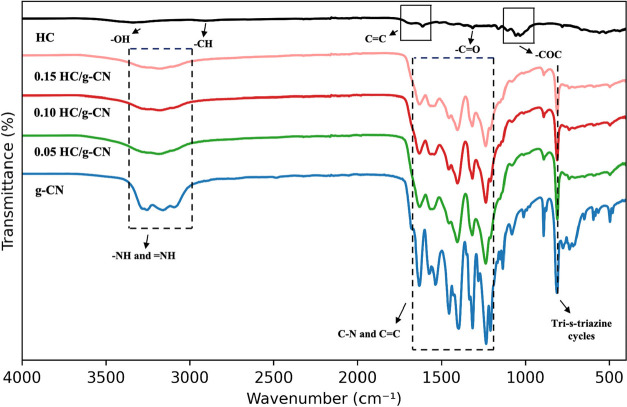
FTIR spectra of HC, g-CN, and HC/g-CN with different loading.

The chemical composition and surface functional groups of the 0.10 HC/g-CN composite were analyzed by XPS, and the results are presented in Figure 10. The XPS survey spectrum (Figure 10a) demonstrated the presence of C, N, and O as the main elements. The atomic percentages of C, N, and O were determined to be 40.68%, 56.15%, and 3.16%, respectively. High-resolution C 1s spectra (Figure 10b) were deconvoluted into two main peaks located at 288.22 and 285.13 eV. The peak at 285.13 eV was assigned to sp^2^-hybridized C–C/C=C bonds, which could be attributed to amorphous carbon. The dominant peak at 288.22 eV was attributed to sp^2^-hybridized carbon present in N–C=N bonds, which were associated with the triazine rings of g-CN.^68^ In N 1s spectrum (Figure 10c), four characteristic peaks were observed at 398.7, 401.35, 400.11, and 404.5 eV. These peaks correspond to sp^2^-hybridized nitrogen in C=N–C, tertiary nitrogen atoms in N–(C)^3^ groups, amino functional groups C–N–H from incomplete condensation, and π-excitation, respectively.^10^ The O 1s spectrum (Figure 10d) exhibited three distinct peaks at 532.58, 536.07, and 531.57 eV. The lower binding energy peak at 531.57 eV was ascribed to C=O functional groups (carbonyl oxygen), possibly originating from the surface oxygenated species of HC. The dominant peak at 532.58 eV is attributed to C–O–C bonds, which indicate possible ether linkages between HC and g-CN. The peak at 536.07 eV is ascribed to oxygen in a highly electron-deficient chemical environment, which is consistent with weakly bound oxygen-containing surface species like CO_2_ or H_2_O.^34,75^

**Figure 10:**
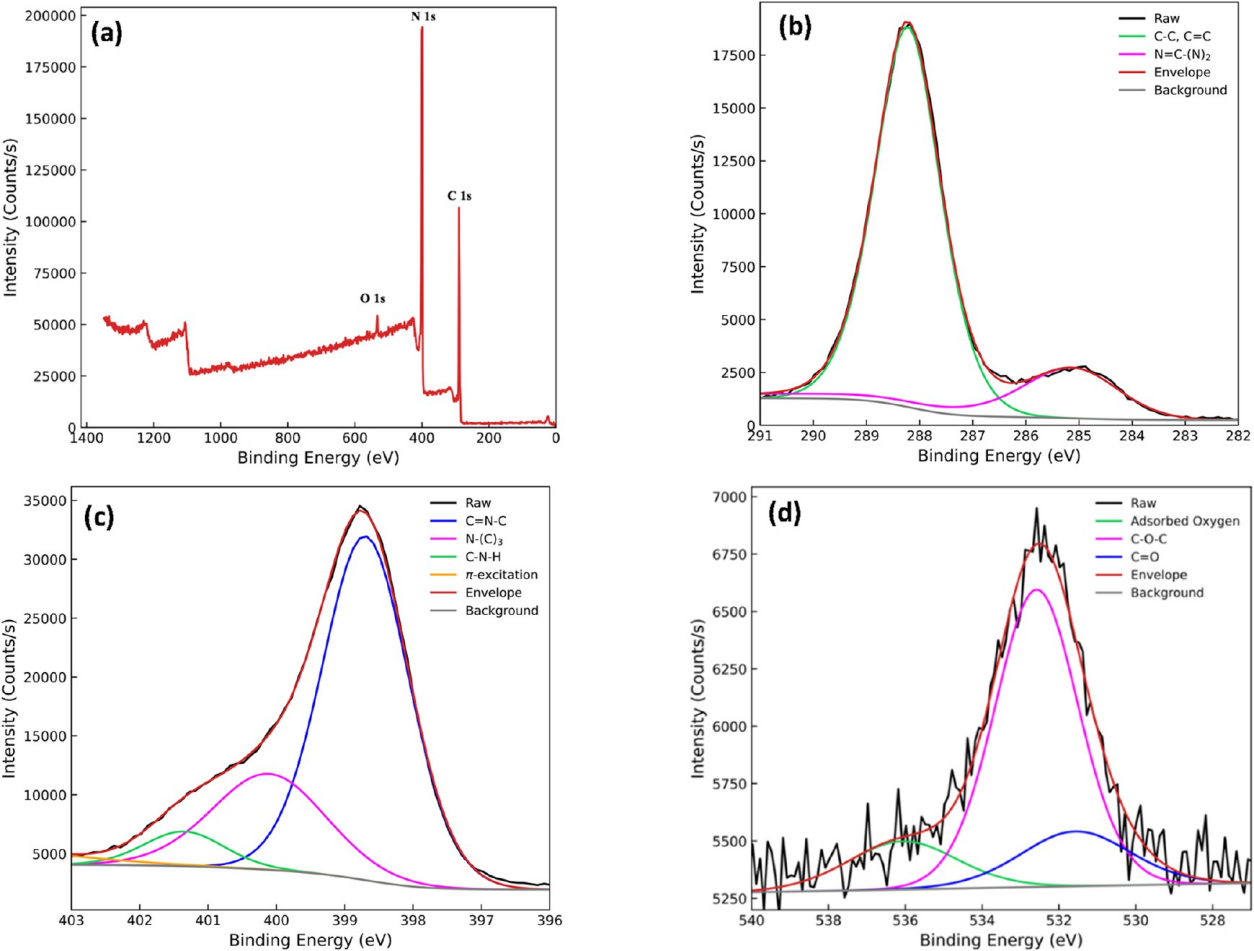
(a) XPS survey spectra of 0.10 HC/g-CN, and high resolution XPS (b) C 1s, (c) N 1s and (d) O 1s spectra of 0.10 HC/g-CN.

#### 3.4.3 Optical Properties of HC/g-CN

The optical absorption properties of the photocatalysts were evaluated by UV–vis spectroscopy (Figure 11a). Pure g-CN and HC/g-CN exhibited a strong absorption edge up to 450 nm, which corresponds to the electron transitions from the VB populated by N 2p to CB formed by C 2p orbitals, which is characteristic of its semiconductor structure.^76^ The addition of HC into g-CN resulted in a moderate increase in absorption across the measured wavelength range, with a slight broadening of the absorption tail into the visible region, indicating improved visible light harvesting through increasing background absorption rather than a significant shift in the absorption edge. The 0.05 HC/g-CN sample exhibited the highest absorption intensity, suggesting that excessive HC can lead to light scattering that reduces optical efficiency. The optical band gap (*E*_g_) of the photocatalysts was determined using the Tauc plot, which involves plotting (α*h*ν)^1/2^ against *h*v for materials exhibiting an indirect band gap, where α denotes the absorption coefficient, *h* is Planck’s constant, and ν represents the photon frequency. As shown in the Tauc plots (Figure 11b), the band gap energy decreased from 2.66 eV for pure g-CN to 2.27, 2.14, and 2.07 eV for the 0.15, 0.10, and 0.05 HC/g-CN composites, respectively. The moderate reduction in band gap contributes to improved visible-light utilization, though this alone does not fully account for the enhanced photocatalytic performance observed. More importantly, by functioning as a conductive electron-transfer medium, HC inhibits charge carrier recombination and extends the lifetime of photogenerated electron–hole pairs, consistent with the PL results presented in Figure 12.^34^

**Figure 11:**
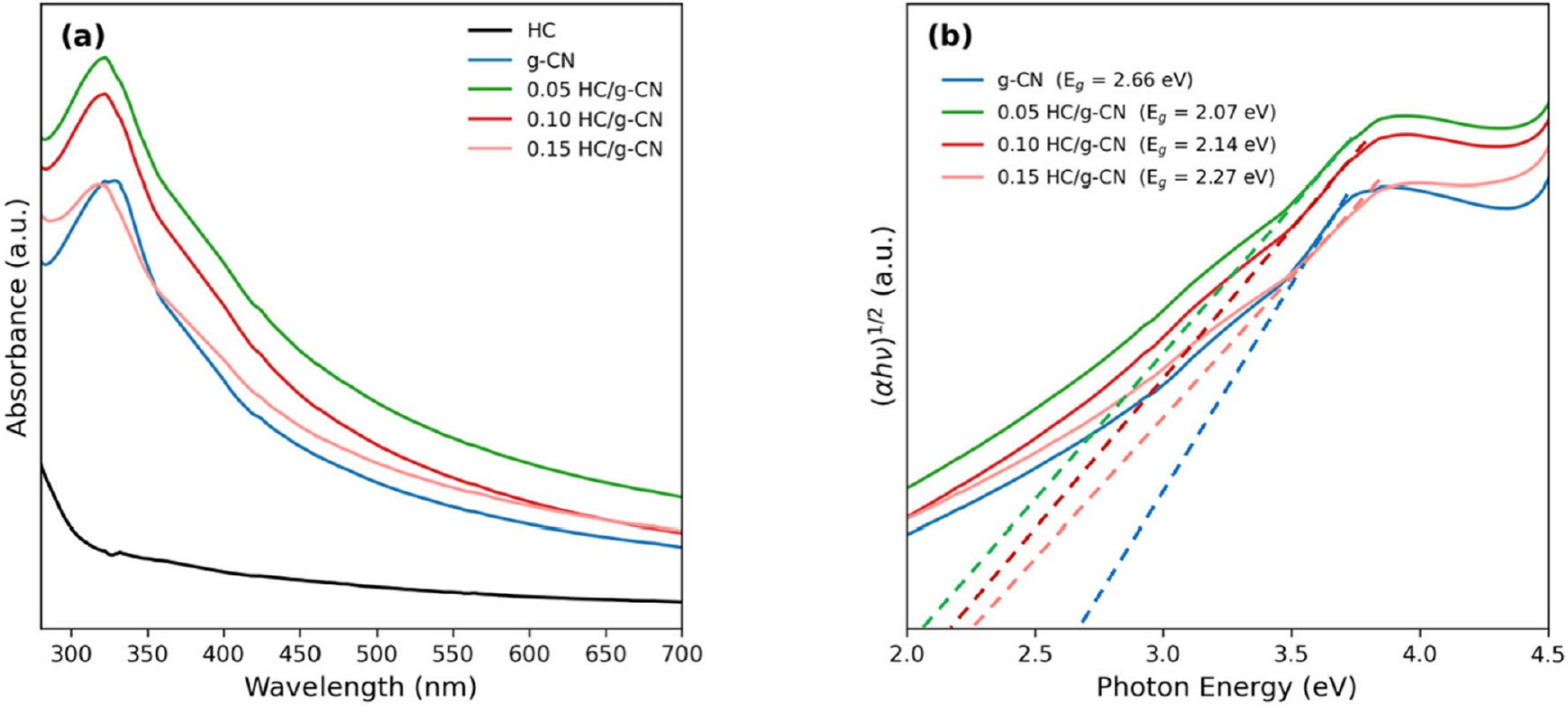
(a) UV–vis spectra and (b) Tauc plots of pure g-CN and HC/g-CN composites with different HC loadings.

**Figure 12:**
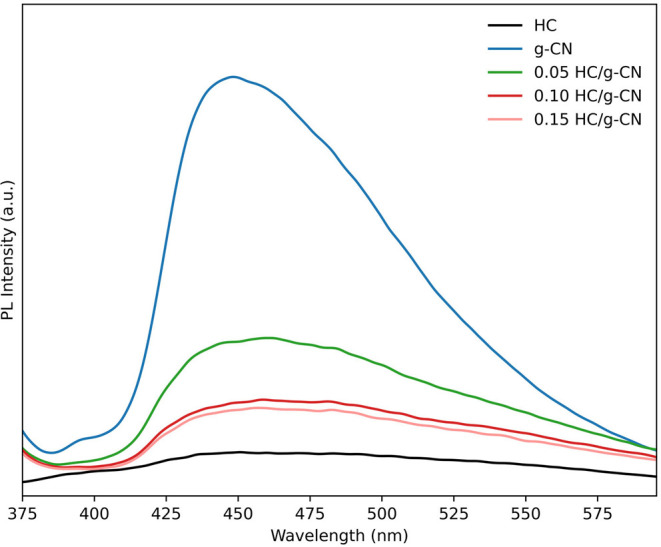
PL spectra of g-CN and HC/g-CN with different HC (0.05, 0.10, and 0.15) loading.

The separation efficiency of photogenerated charge carriers in g-CN and HC/g-CN photocatalysts was evaluated by PL spectroscopy at 350 nm excitation wavelength, as illustrated in Figure 12. All samples exhibited a similar emission profile with a dominant peak centered around 450 nm. Compared to pure g-CN, the PL intensity of HC/g-CN composites decreased with increasing HC content, revealing improved charge separation facilitated interfacial electron transfer between g-CN and HC.^10^ However, it should be noted that a decrease in PL intensity does not necessarily demonstrate improved charge separation, as it may also arise from electron trapping at defect sites or nonradiative recombination pathways, which are often associated with reduced photocatalytic activity.^77–79^ In the present study, the decrease in PL intensity for HC/g-CN composites was accompanied by a slight improvement in TOC removal. This may confirm a positive role of HC in facilitating interfacial charge transfer between HC and g-CN.

### 3.5 Photocatalytic Degradation Studies

The optimization study for photocatalytic degradation was designed using the Box-Behnken method and analyzed with Minitab statistical software. Photocatalytic degradation experiments were performed using real sugar beet factory wastewater, with TOC removal efficiency as the response variable. The effects of HC/g-CN composite amount (g, per 20 g urea), catalyst dosage (g/L), pH, and H_2_O_2_ concentration (mM) were examined. The uncoded levels of these factors and the corresponding experimental results for photocatalytic degradation are presented in Table 4.

**Table 4:** Uncoded Values of Factors and the Corresponding Experimental Results for Photocatalytic Degradation

HC added (g)	Catalyst Loading, g/L	pH	H_2_O_2_ Concentration, mM	TOC Removal, %
0.10	1.0	4	10	5.44
0.10	0.6	6	10	8.18
0.10	0.6	6	10	7.32
0.10	0.6	8	20	11.06
0.15	0.6	6	20	6.04
0.10	0.2	4	10	7.48
0.05	0.2	6	10	10.57
0.10	0.2	6	20	7.09
0.10	0.6	6	10	5.89
0.10	0.6	6	10	7.62
0.05	0.6	6	20	10.62
0.05	1.0	6	10	6.12
0.10	0.2	8	10	7.51
0.10	0.6	6	10	7.80
0.15	1.0	6	10	4.80
0.05	0.6	6	0	10.50
0.10	1.0	6	0	7.69
0.10	0.6	4	20	9.67
0.10	0.2	6	0	7.53
0.15	0.6	4	10	6.50
0.05	0.6	4	10	5.40
0.10	1.0	8	10	9.26
0.10	0.6	6	10	8.06
0.15	0.6	8	10	5.97
0.10	0.6	4	0	7.06
0.10	1.0	6	20	9.52
0.15	0.6	6	0	5.90
0.10	0.6	8	0	8.11
0.05	0.6	8	10	10.37
0.15	0.2	6	10	5.80

#### 3.5.1 Effects of Process Parameters on TOC Removal

As shown in Table S5, the overall model for TOC removal was statistically significant (*F* = 3.61, *p* = 0.009), indicating a meaningful correlation between the tested factors and TOC degradation efficiency. Among the linear terms, the HC amount in the HC/g-CN composite (*p* < 0.001) had the most significant effect, followed by pH (*p* = 0.020), while catalyst dosage and H_2_O_2_ concentration did not show linear contributions (*p* > 0.05). Regarding the quadratic terms, the H_2_O_2_ concentration had a statistically significant effect (*p* = 0.028), which suggested a nonlinear relationship between oxidant amount and degradation efficiency. In contrast, other quadratic terms were found to be statistically insignificant (*p* > 0.3). Among the two-way interaction terms, only the interaction between HC ratio and pH was statistically significant (*p* = 0.036), demonstrating that the catalyst’s performance is sensitive to pH-dependent surface interactions. Other interactions were not statistically significant. The coefficient of determination (*R* ^2^ = 0.7710) confirmed that the model explained a substantial portion of the variation in TOC removal. While this value is moderate compared to degradation-based responses, this is expected for mineralization, as TOC removal is inherently more complex than pollutant degradation due to involvement of multiple sequential oxidation steps and intermediate species generation. The statistically significant overall model (*p* = 0.009) confirms the adequacy of the model within the studied experimental domain. Experimental reproducibility was assessed through six center point replicates, yielding a pure error mean square of 0.699 (df = 5). The lack-of-fit test (*F* = 2.54, *p* = 0.158) confirmed adequate model fit and experimental reliability. Control experiments conducted in the absence of both light and H_2_O_2_ showed that TOC removal was limited to 2.80% using 0.10 HC/g-CN, while experiments under center point conditions without light resulted in 4.47% removal, confirming that photocatalytic oxidation rather than adsorption was the primary degradation mechanism. The Pareto chart (Figure S3a) presents the standardized effects of each factor and their interaction on TOC removal. Among the linear terms, HC amount (A) showed the highest standardized effect, exceeding the significance threshold, while pH (C) and the quadratic term for H_2_O_2_ (DD) demonstrated borderline contributions consistent with the ANOVA results. The remaining terms fell below the reference line, which is likely due to the complex nature of real wastewater, where various components interfere and reduce the resolution of individual parameters. The normal probability plot (Figure S3b) further supported the model’s validity by confirming that the residuals were approximately normally distributed since it showed a linear trend, with a slight S-shaped deviation at the tails, as also supported by the lack-of-fit result.

A second-order regression model based on a Box-Behnken design was employed to optimize TOC removal, accounting for the interactive effects of HC amount, catalyst loading, pH, and H_2_O_2_ concentration (Figure 13). As shown in Figure 13a, low HC additions improved removal efficiency, increasing the amount after a certain level resulted in a remarkable decline, which may be attributed to excessive surface coverage or raised light scattering, which can hinder photocatalysis activity. Furthermore, catalyst loading caused a nonlinear effect on TOC removal (Figure 13c); increasing the loading to 0.81 g/L slightly enhanced efficiency because more active sites were available. This phenomenon can be explained by the light-shielding effect caused by excessive catalyst accumulation, which restricts photon penetration and reduces photocatalytic efficiency. Consequently, the optimal catalyst amount depends on both the photocatalytic system and the characteristics of the pollutants. Also, the concentration of H_2_O_2_ showed a nonlinear effect on TOC removal, similar to that of catalyst loading (Figure 13d). The incorporation of H_2_O_2_ into the heterogeneous photocatalytic system enhanced the degradation of sugar factory wastewater by acting as an effective electron scavenger, thereby facilitating charge separation and promoting the generation of hydroxyl radicals. Elevating the pH of sugar factory wastewater from acidic to alkaline conditions significantly improved TOC removal efficiency under visible light irradiation (Figure 13a). The degradation occurred more effectively in alkaline media, and this enhancement can be attributed to two principal factors: (i) at low pH, the tendency of HC/g-CN particles to agglomerate can hinder light absorption; (ii) excess H^+^ ions under acidic conditions may interact with electron-rich functional groups in organic pollutants, reducing their reactivity toward electrophilic hydroxyl radicals.^80^ On the other hand, the model predicted the optimal conditions for maximizing TOC removal, as shown by the star marker in Figure 13, as 0.05 g HC, 0.81 g/L catalyst loading, pH 8, and 20 mM H_2_O_2_. Under these conditions, the TOC removal efficiency was estimated to reach approximately 13.00%. However, experimental validation under the same conditions resulted in a slightly higher TOC removal of 14.02%.

**Figure 13:**
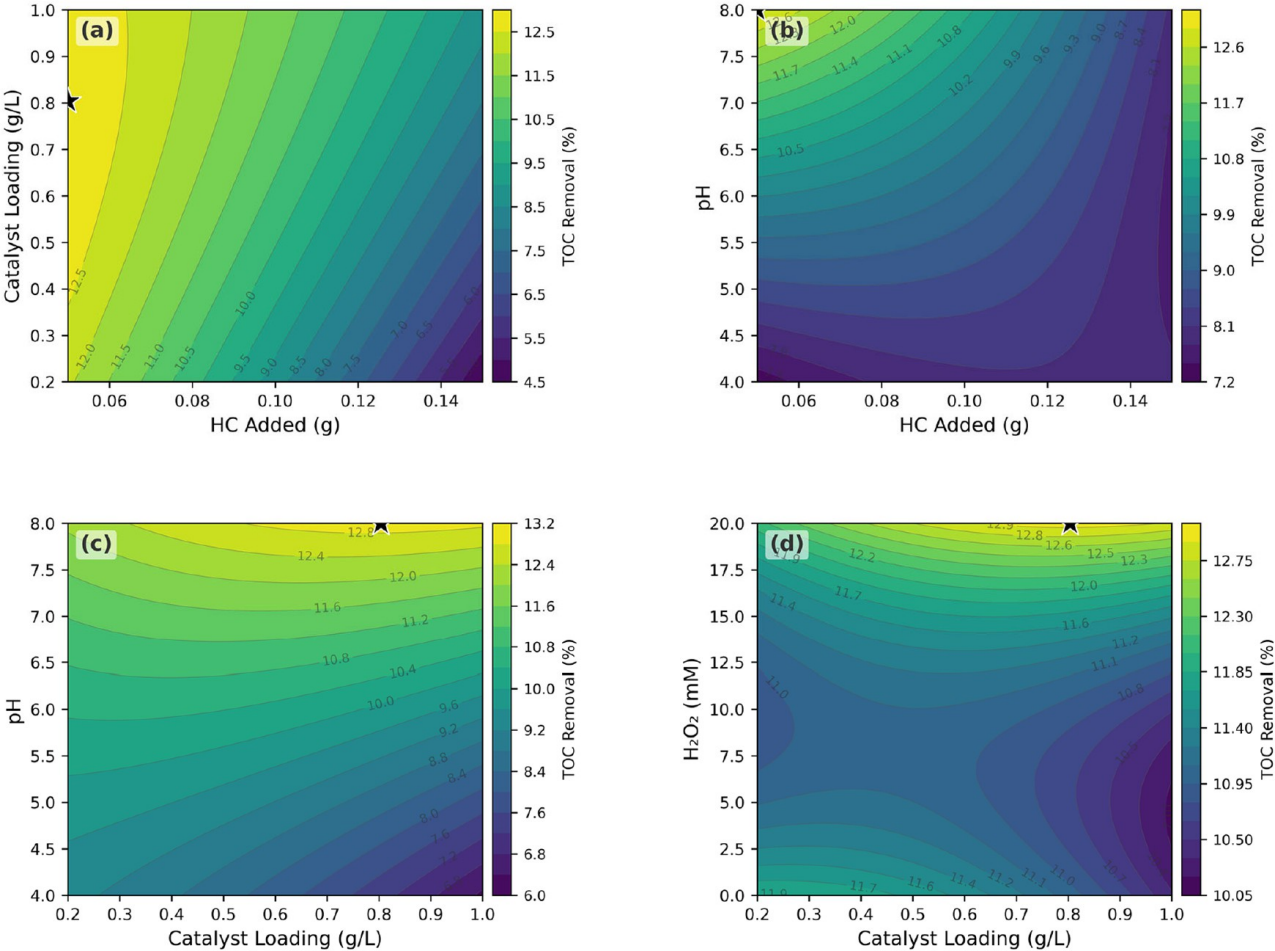
Response surface contour plots showing the effects of (a) HC amount and catalyst loading, (b) HC amount and pH, (c) catalyst loading and pH, and (d) catalyst loading and H_2_O_2_ concentration on TOC removal efficiency (other variables fixed at optimum).

Although the maximum TOC and COD removal achieved in this study were 14.02% and 23.94%, respectively, using 0.8 g/L 0.05 HC while pH equals 8 in the presence of 20 mM H_2_O_2_, it was important to consider the exceptionally high initial TOC concentration of the real sugar factory wastewater used (>7000 mg/L). Compared to model solutions or synthetic wastewater commonly used in the literature, which often have TOC levels below 500 mg/L, treating such concentrated effluents posed a significant challenge. In this context, it was diluted 1:10 v/v and tested under optimal conditions to assess the effect of sugar factory wastewater concentration. The outcomes showed that the concentration was highly effective for TOC and COD removal, as photocatalytic treatment of diluted wastewater resulted in higher removals (18.9% and 38.12%, respectively).

The observed limited mineralization reflects a widely known photocatalytic mechanism that proceeds through the generation of hydroxyl (−OH) and superoxide (O_2_^–^) radicals, which initiate oxidative breakdown of organic structures. However, in real industrial wastewaters, these radicals are consumed not only by target organic compounds but also by inorganic and ionic species, which act as competitive −OH scavengers, such as carbonates, bicarbonates, sulfates, and chlorides. The resulting anionic radicals are far less efficient at mineralizing complex organic fractions because they possess substantially lower oxidation potentials than −OH.^81–83^ Zafar et al. demonstrated that TOC removal dropped from 66.7% in deionized water to just 15% in surface water and 28% in seawater using Fe-TiO_2_ photocatalysis, with individual ion additions of carbonate and sulfate reducing TOC removal to 20% by direct −OH scavenging. The sugar factory wastewater used in this study contains all of these ionic species simultaneously at process-relevant concentrations, making competitive radical consumption and inevitable limitation.^81^

The decoupling between target and compound degradation and bulk TOC mineralization is well-established in the literature. Pino-Sandoval et al. demonstrated a solar TiO_2_P25 photocatalyst that achieved complete or near-complete pharmaceutical degradation in real hospital wastewater at pH 8.1, yet TOC reduction reached only 18.1% at 400 kJ/m^2^ UV energy, attributable to the scavenging action of inorganic ions and dissolved organic matter present in the effluent matrix.^82^ Similarly, Pirilä et al. treated an industrial pharmaceutical wastewater with 7600 mg/L TOC using TiO_2_P25 under UV-A irradiation, and reported approximately 20% TOC removal after 3 h, with degradation rate constants reduced by factors of 4–12 compared to distilled water due to competition for catalyst active sites.^84^ Samanamud et al. obtained a maximum effective TOC degradation of only 14.23% when applying solar ZnO photocatalysis to real dairy wastewater at pH 8 over 3 h, and noted that photocatalytic treatment improved BOD/COD ratio from 0.368 to 0.431, indicating enhanced biodegradability despite modest mineralization.^83^ Moreira et al., further illustrated this principle by demonstrating that despite near-complete removal of nine organic micropollutants from real urban WWTP effluent within 10 min using g-CN photocatalytic process under visible LED irradiation, no significant change in the dissolved organic carbon (DOC) content of the effluent was detected, attributing this to the orders -of-magnitude disparity between the micropollutant concentrations and the background dissolved organic carbon pool, which constitutes the dominant carbon sink in real matrices.^85^ Furthermore, Tekin et al. reported TOC removal from 3.78% to 26.89% by changing the catalyst loading (Cu–BiOI) from 0 to 8 g/L at pH 7, using a 1.8 g/L sucrose solution (initial TOC, 712 mg/L) with 100 mM H_2_O_2_ for a 1 h treatment time.^86^ Also, Kee et al. reported that increasing wastewater concentration decreased COD removal efficiency during photocatalytic degradation.^87^ It can be explained by the fact that the wastewater matrix significantly affects photocatalytic efficiency due to the presence of competing organic and inorganic components, as Pirilä et al. confirmed that real industry wastewater matrices negatively affect pollutant removal efficiency relative to synthetic solutions.^81,84^ In a representative comparison, complete degradation of target pharmaceuticals in distilled water yielded 70% TOC removal, whereas the identical treatment applied to real hospital wastewater produced only 18.1% TOC removal despite complete drug elimination, directly attributed to the consumption of reactive oxygen species by the effluent organic matter matrix.^82^ Moreover, Quiñones et al. demonstrated at pilot scale that even a combined solar photocatalytic ozonation system applied to biologically pretreated WWTP secondary effluent achieved less than 35% TOC removal after 5 h, while simplifier TiO_2_/solar photocatalysis alone reached only 8%, confirming that incomplete mineralization under practical advanced oxidation conditions is the expected outcome when treating real, complex aqueous matrices.^88^ Table 5 provides a comparison of the photocatalytic performance achieved in this study with previous studies in the literature, which include different operating conditions on real industrial and complex wastewaters.

**Table 5:** Comparison of Photocatalytic TOC Removal Performance in Real Wastewater Systems

Catalyst	Wastewater	TOC removal (%)	Conditions	refs
Fe–TiO_2_	Surface water	15	35 mL reaction cell, 180 min	^81^
TiO_2_ (P25)	Hospital wastewater	18.1	1.0 g/L catalyst, 400 kJ m^–2^ UV	^82^
TiO_2_ (P25)	Industrial wastewater	∼20	Batch reactor, varied pH and loading	^84^
ZnO (immobilized)	Dairy wastewater	14.23	pH 8, 180 min reaction	^83^
g-CN	Real WWTP effluent	no significance changes (DOC)	LED irradiation (417 nm), batch reactor	^85^
TiO_2_/Photo-ozonation	WWTP secondary effluent	35	Pilot-scale CPC reactor, O_3_ + light	^88^
HC/g-CN	Real sugar industry wastewater	14.02	0.81 g/L catalyst, pH 8, 20 mM H_2_O_2_	This work

#### 3.5.2 Kinetic Study

The study used a pseudohomogeneous first-order kinetic model to describe the photocatalytic degradation mechanism. As illustrated in Figure 14a, the reaction showed two phases: an initial rapid stage with a calculated activation energy of 6.87 kJ/mol, attributed to easily oxidizable compounds, and a slower stage with an activation energy of 19.97 kJ/mol, associated with more persistent organics. This behavior is often encountered in heterogeneous photocatalytic systems, especially when complex organic mixtures are treated. The calculated rate constants were summarized in Table 6. Activation energies were derived from an Arrhenius plot corresponding to each phase (Figure 14b). These values align with previously reported activation energies for TOC degradation in similar photocatalytic systems, such as 14.44 and 27.2 kJ/mol.^86^

**Figure 14:**
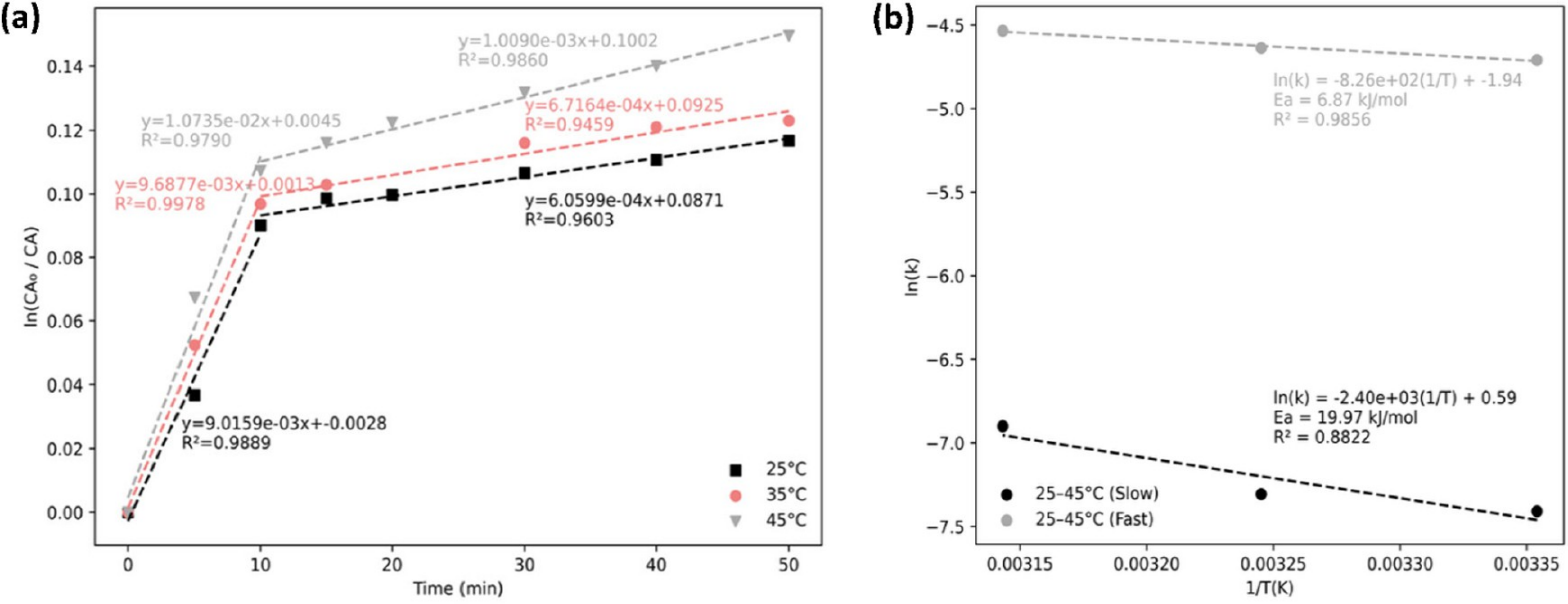
(a) Pseudohomogeneous first-order two-step reaction model obtained by using 0.05 HC/g-CN (b) Arrhenius plot.

**Table 6:** Photocatalytic Rate Constants Obtained by Using 0.05 HC/g-CN

	Fast step		Slow step	
Temperature (°C)	Rate constant × 10^3^ (min^–1^)	*R* ^2^	Rate constant × 10^4^ (min^–1^)	*R* ^2^
25	9.016	0.989	6.060	0.960
35	9.688	0.998	6.716	0.946
45	10.735	0.979	10.090	0.986

#### 3.5.3 Photocatalytic Reaction Mechanism

The radical scavenging experiments were conducted under optimized conditions using EDTA (1 mM) as a hole (h^+^) scavenger, isopropanol (IPA, 10 mM) as a hydroxyl radical (^•^OH) scavenger, and l-ascorbic acid (1 mM) as a superoxide radical (^•^O_2_^–^) scavenger to identify the dominant reactive species. As shown in Figure 15a, the addition of l-ascorbic acid caused the most significant decrease in TOC removal (0.60%), indicating that ^•^O_2_^–^ plays the dominant role in the photocatalytic degradation of sugar factory wastewater using HC/g-CN. The addition of IPA also resulted in a notable decrement (2.20%), demonstrating a considerable contribution of ^•^OH radicals to the process. In contrast, EDTA showed a minor effect (12.06%), suggesting that h^+^ plays a negligible role under the tested conditions. Based on these results, the proposed photocatalytic mechanism of HC/g-CN under visible light is as follows: upon light absorption, HC/g-CN generates electron–hole pairs. Photogenerated electrons reduce dissolved O_2_ to ^•^O_2_^–^, while holes oxidize surface-adsorbed H_2_O or OH to produce ^•^OH. H_2_O_2_ promotes ^•^OH generation by acting as an electron scavenger, consistent with similar systems reported in the literature, where ^•^O_2_^–^ was also identified as the dominant reactive species.^89^ Based on the results, a photocatalytic mechanism dominated by ^•^O_2_^–^ radicals and involving partial oxidation pathways is proposed and illustrated in Figure 15b.

**Figure 15:**
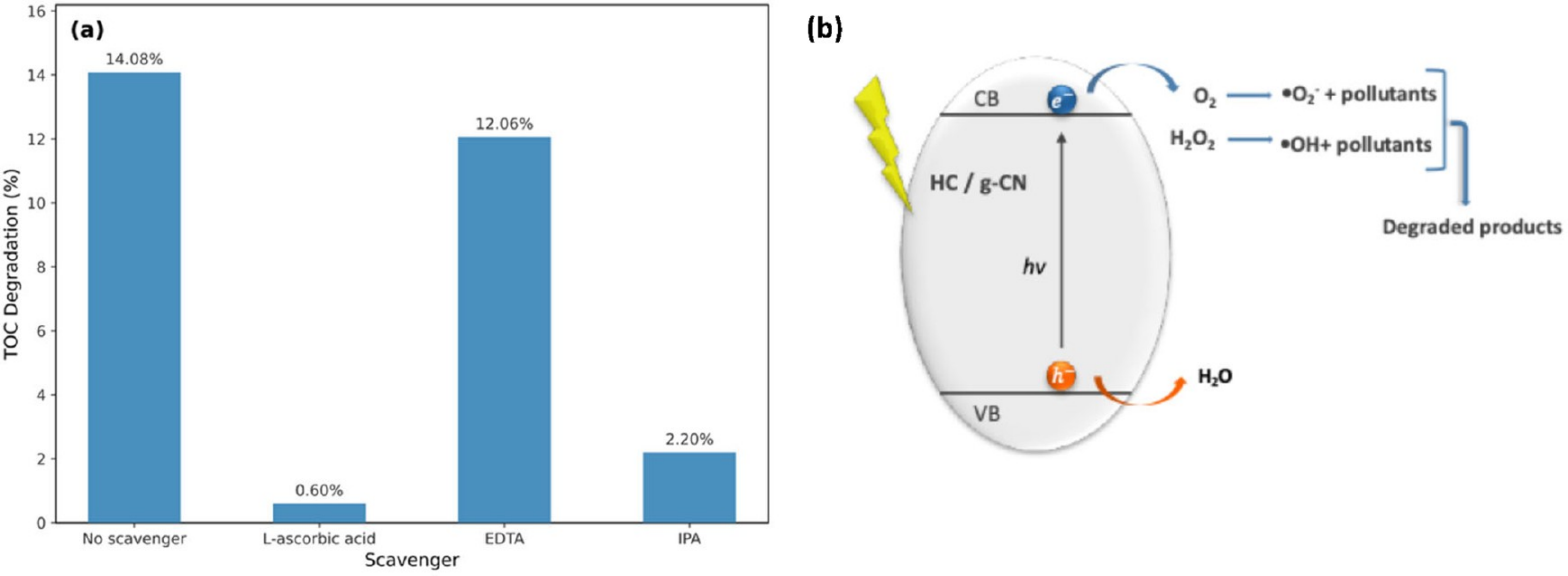
(a) Effect of radical scavengers on TOC removal efficiency of 0.05 HC/g-CN under optimized conditions. (b) Proposed photocatalytic degradation mechanism of HC/g-CN under visible light irradiation.

#### 3.5.4 Reusability

Under the optimized reaction conditions, the spent 0.05 HC/g-CN catalyst was recovered and reused for five consecutive cycles to evaluate its reusability (Figure 16a). The TOC degradation efficiency decreased gradually from 14.08% in the first cycle to 9.51% in the fifth cycle, demonstrating reasonable stability over repeated use. The improved cycling stability of the HC/g-CN composite can be attributed to HC’s role as a carbonaceous support, which facilitates electron transfer and stabilizes the composite structure, consistent with biomass-derived carbon/g-CN systems reported in the literature.^27^ Moreover, postreaction XRD analysis confirmed the retention of characteristic g-CN diffraction peaks after successive cycles at pH 8, implying that the catalyst structure was not significantly altered under the tested conditions (Figure 16b). The emergence of minor new peaks is attributed to the deposition of constituents from the wastewater. SEM images revealed gradual morphologic changes consistent with surface fouling over repeated cycles (Figure 16c–e). Figure 16d,16e display metal-rich aggregates formed from the wastewater, which contains high levels of Fe (8.988 mg/L), Al (839.9 μg/L), Mn (447.9 μg/L), and Zn (352.9 μg/L), as indicated in Table S2. Catalyst mass loss of 20% was observed by 5 cycles, which, alongside surface fouling, contributed to a gradual decline in activity. For comparison, pure g-CN showed more pronounced efficiency decline over three cycles (16.73% to 9.54%), highlighting the staining role of HC incorporation (Figure S6).

**Figure 16:**
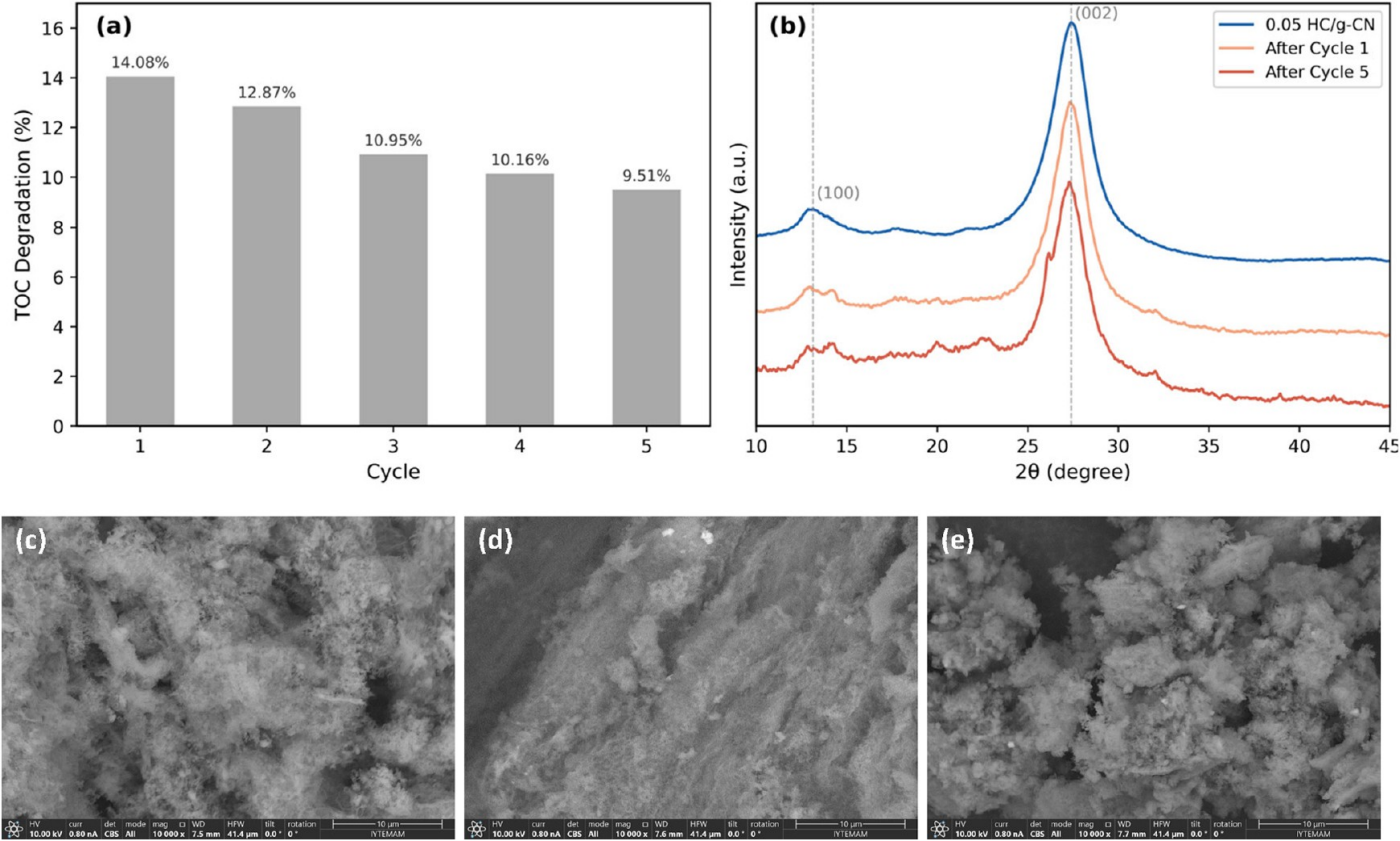
(a) TOC degradation efficiency of 0.05 HC/g-CN over 5 cycles, (b) XRD patterns before and after use, and (c–e) SEM images of fresh catalyst, spent catalyst after 3rd cycle, and spent catalyst after 5th cycle.

## 4 Conclusion

The study investigated the use of hydrothermally carbonized sugar beet pulp as a support material for synthesizing g-CN photocatalysts. A Box-Behnken design was employed to optimize both the HTC conditions and the photocatalytic reaction parameters, enabling exploration of the multiparameter space with a reduced number of experiments. The optimization revealed that biomass amount and temperature were the dominant factors for HC yield, with the highest predicted yield of 36.74% achieved at 20 g biomass, 200 °C, and 60 min. For the photocatalytic process, HC amount and pH were identified as the most influential parameters, with the optimal conditions (0.05 g HC, 0.81 g/L catalyst loading, pH 8, 20 mM H_2_O_2_) yielding 14.02% TOC removal from real sugar factory wastewater. The properties of hydrochar were mainly influenced by the temperature and the amount of biomass used during processing. Characterization showed that HC had improved aromatization, graphitization, and thermal stability compared to untreated SBP. When incorporated into g-CN, the HC improved the surface area of the resulting composite and functioned as a conductive electron-transfer medium, inhibiting charge carrier recombination and extending the lifetime of photogenerated electron–hole pairs. The composite material was effective in removing organic pollutants from real sugar factory wastewater and showed greater stability and reusability than pure g-CN. Overall, the research demonstrates that hydrochar derived from agricultural waste can serve as a sustainable support for g-CN photocatalysts, thereby improving their performance in wastewater treatment. Despite the promising results, the moderate TOC removal efficiencies achieved reflect the high organic loads of real industrial wastewaters, which limit radical utilization and mineralization efficiency. Future work should focus on improving removal performance through catalyst modification and integration with complementary treatment processes. The finding highlights a circular economy approach, as the solid waste of the sugar industry is valorized as a photocatalytic support material for treating the liquid waste of the same facility. However, scale-up presents challenges, including efficient catalyst recovery due to the powdered nature of the catalyst, and reactor design to ensure adequate light penetration and distribution.

## Supplementary Material



## Data Availability

The data sets generated during and/or analyzed during the current study are available within the manuscript and its Supporting Information files.

## Notes

GC-MS analysis of sugar factory wastewater (Table S1); elemental composition of wastewater (Table S2); ANOVA results for hydrochar yield and TOC removal (Tables S3 and S5); ultimate and proximate analysis of hydrochar samples (Table S4); and additional figures including Pareto charts, FTIR, XRD, TG/DTG curves, and catalyst reusability and postcharacterization results (Figures S1–S6) (PDF)

## Abbreviations

Adj: Adjusted
BET: Brunauer-Emmet-Teller
BJH: Barrett–Joyner–Halenda
CB: Conduction Band
COD: Chemical Oxygen Demand
DF: Degree of Freedom
DLS: Dynamic Light Scattering
DOC: Dissolved Organic Carbon
DTG: Derivative Thermogravimetry
EDX: Energy-Dispersive X-ray
FC: Fixed Carbon
FTIR: Fourier Transformed Infrared Spectroscopy
GC-MS: Gas Chromatography–Mass Spectrometry
g-CN: Graphitic Carbon Nitride
H_2_O_2_: Hydrogen Peroxide
HC: Hydrochar
HCl: Hydrochloric Acid
HHV: Higher Heating Value
HTC: Hydrothermal Carbonization
HTL: Hydrothermal Liquefaction
ICP-MS: Inductively Coupled Plasma Mass Spectrometry
NaClO_2_: Sodium Chlorite
NaOH: Sodium Hydroxide
PL: Photoluminescence
SBP: Sugar Beet Pulp
SEM: Scanning Electron Microscopy
TG: Thermogravimetry
TGA: Thermogravimetric Analysis
TN: Total Nitrogen
TOC: Total Organic Carbon
UV–vis: UV–visible
VB: Valence Band
VM: Volatile Materials
XPS: X-ray Photoelectron Spectroscopy
XRD: X-ray Diffraction
