# Supplementary material for: Photocatalytic Treatment of Real Sugar Industry Wastewater Using Lignocellulosic Biomass-Derived Hydrochar/g-CN
Source: ACS Omega. 2026 Jun 16;11(25):37680–99. doi: 10.1021/acsomega.6c02485 (PMC13325150; doi:10.1021/acsomega.6c02485)
Supplement: Supplementary file 1 [file ao6c02485_si_001.pdf]

Photocatalytic Treatment of Real Sugar Industry Wastewater Using Lignocellulosic Biomass-Derived Hydrochar/g-CN

Bahriyenur Arabacı[1], Aslı Yüksel[1*], Canan Varlıklı[2]

[1]*Izmir Institute of Technology, Department of Chemical Engineering, 35430, Urla, Izmir, Turkiye*

[2]*Izmir Institute of Technology, Department of Photonics, 35430, Urla, Izmir, Turkiye*

asliyuksel@iyte.edu.tr

Table S1. Table 4. GC-MS results of sugar factory wastewater.

RT (min)	Area%	Compound
**0.959**	1.97	Hexane
**1.051**	3.25	2-Pentanamine, 4-methyl-
**1.176**	2.87	Hexane
**3.488**	2.92	Ethyl alcohol
**6.438**	34.83	1,2-Propanediol
**10.247**	2.50	2-Propanone, 1-hydroxy-
**12.599**	19.88	Acetic acid
**12.757**	1.37	Methane, dipropoxy-
**14.024**	3.43	Propanoic acid
**14.662**	1.32	2,3-Butanediol
**15.857**	4.85	Butanoic acid
**23.772**	7.01	Ethanol, 2,2'-oxybis-
**26.649**	9.63	Triethylene glycol
**27.595**	4.18	4H-Pyran-4-one, 2,3-dihydro-3,5-dihydroxy-6-methyl-

Table S2. The elemental analysis results of sugar factory wastewater.

Element	Concentration (mg/L or µg/L)
**B (µg/L)**	98.53
**Na (mg/L)**	62.63
**Mg (mg/L)**	13.73
**Al (µg/L)**	839.9
**K (mg/L)**	324.0
**Ca (mg/L)**	338.4
**Cr (µg/L)**	10.14
**Mn (µg/L)**	447.9

**Fe (mg/L)**	8.988
**Co (µg/L)**	9.270
**Ni (µg/L)**	58.43
**Cu (µg/L)**	96.40
**Zn (µg/L)**	352.9
**As (µg/L)**	8.536
**Se (µg/L)**	2.230
**Cd (µg/L)**	0.1650
**Sb (µg/L)**	1.091
**Pb (µg/L)**	9.592
**TN (N) (mg/L)**	312.0

Table S3. ANOVA results for hydrochar yield.

Source	DF	Adj SS	Adj MS	F-Value	P-Value
**Model**	9	328.873	36.541	21.01	0.000
**Linear**	3	295.501	98.500	56.64	0.000
Amount (g)	1	29.915	29.915	17.20	0.003
Temperature (°C)	1	262.778	262.778	151.11	0.000
Time (min)	1	2.808	2.808	1.61	0.239
**Square**	3	26.147	8.716	5.01	0.030
Amount (g)*Amount (g)	1	0.344	0.344	0.20	0.668

Temperature (°C) *Temperature (°C)	1	17.703	17.703	10.18	0.013
Time (min)*Time (min)	1	10.461	10.461	6.02	0.040
**2-Way Interaction**	3	7.224	2.408	1.38	0.316
Amount (g)*Temperature (°C)	1	0.005	0.005	0.00	0.959
Amount (g)*Time (min)	1	6.943	6.943	3.99	0.081
Temperature (°C) *Time (min)	1	0.276	0.276	0.16	0.701
**Error**	8	13.912	1.739		
**Lack-of-Fit**	3	7.627	2.542	2.02	0.229
**Pure Error**	5	6.285	1.257		
**Total**	17	342.785			

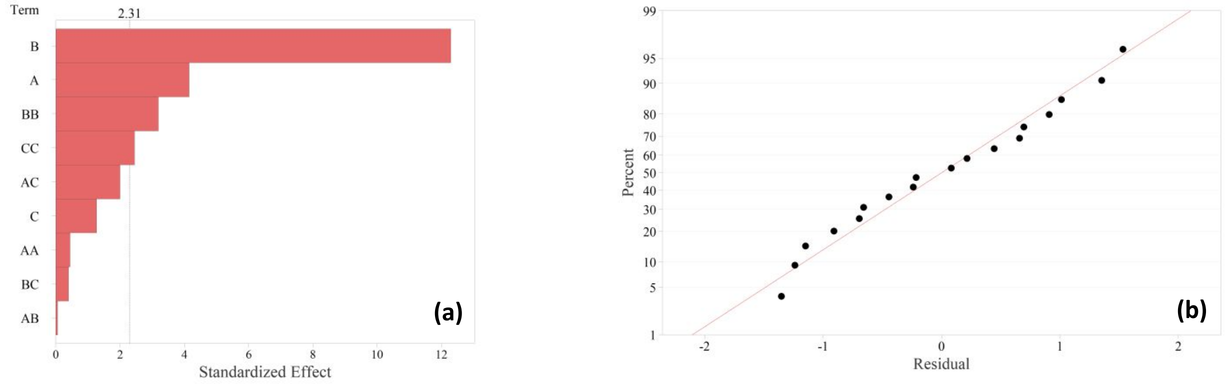

Figure S1. a) Pareto chart of standardized effect (A: amount (g), B: temperature (°C), and C: time (min)), and b) normal probability plot of residuals for hydrochar yield.

Table S4. The ultimate and proximate analysis of HC samples.

Hydrochar Number	Ultimate Analysis (%)				Proximate Analysis (%)				HHV
	C	H	N	O	Moisture	Ash	VM	FC	(MJ/ kg)
1	52.40	5.00	1.43	38.06	3.81	3.11	96.19	0.00	19.57

2	51.16	5.22	1.33	38.20	2.70	4.09	97.30	0.00	19.18
3	51.51	4.56	1.62	39.58	4.25	2.73	95.75	0.00	18.66
4	54.92	4.21	1.49	35.89	2.88	3.49	97.12	0.00	19.66
5	66.85	4.13	3.09	21.88	3.92	4.05	96.08	0.00	25.04
6	57.06	4.79	2.09	31.69	3.74	4.37	96.26	0.00	21.33
7	60.24	5.00	2.15	29.06	4.50	3.55	95.50	0.00	23.14
8	65.97	4.01	2.53	22.65	4.08	4.84	95.92	0.00	24.35
9	58.16	4.78	2.12	32.06	3.97	2.88	96.03	0.00	21.98
10	68.02	3.99	2.89	21.83	3.18	3.27	96.82	0.00	25.46
11	67.99	4.77	2.95	20.07	3.17	4.22	96.83	0.00	26.35
12	54.77	5.33	1.76	34.98	3.11	3.16	96.89	0.00	21.09
13	58.27	5.05	2.12	30.91	3.13	3.76	96.87	0.00	21.29

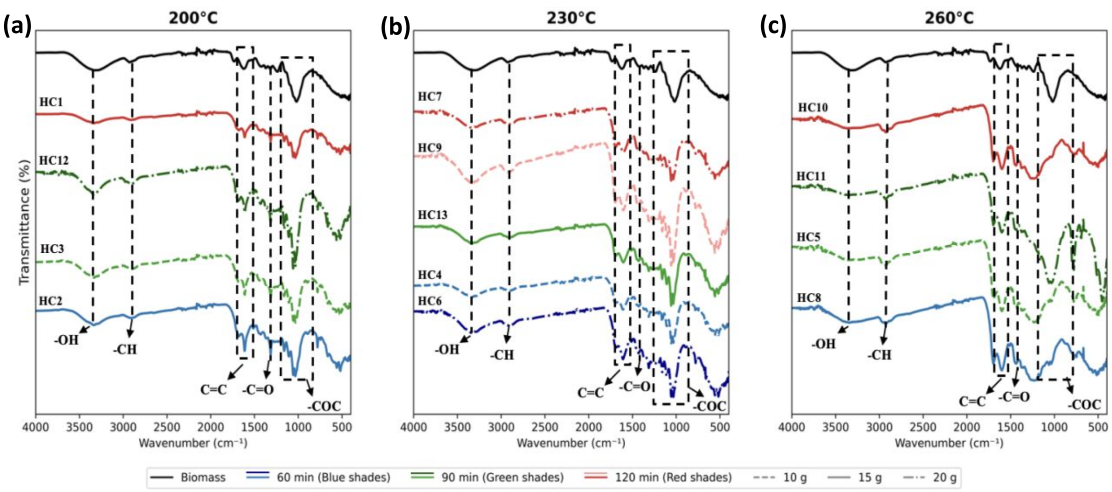

Figure S2. FTIR spectra of HC samples synthesized under different HTC conditions a) 200 °C, b) 230 °C, and c) 260 °C (reaction time: 60-120 min, biomass amount 10-20g).

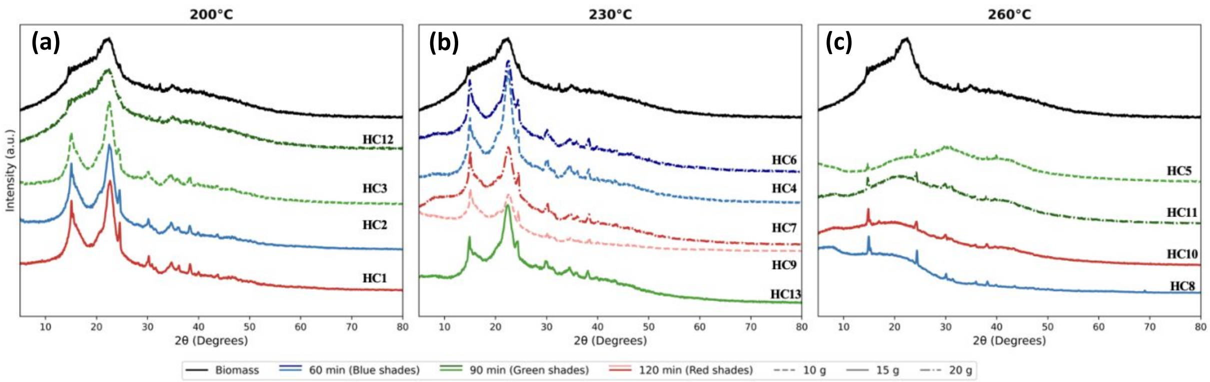

Figure S3. XRD spectra of HC samples synthesized under different HTC conditions a) 200 °C, b) 230 °C, and c) 260 °C (reaction time: 60-120 min, biomass amount 10-20g).

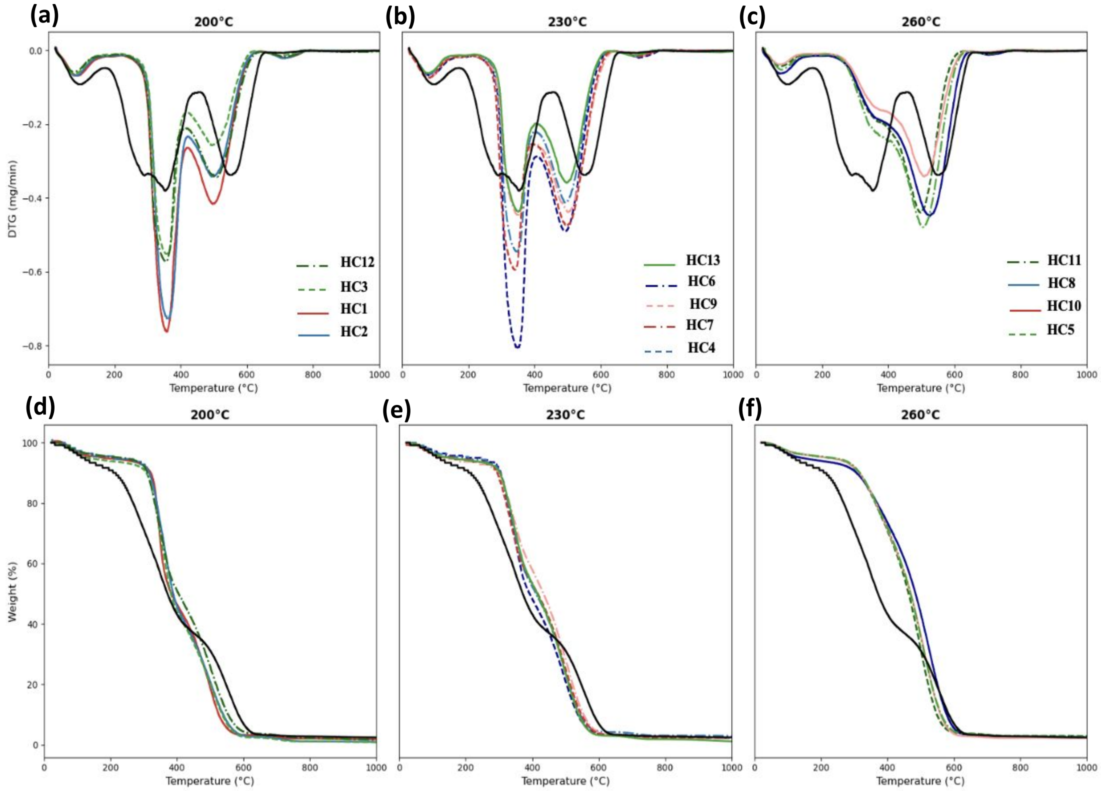

Figure S4. a-c) DTG and d-f) TG curves of HC samples under different HTC conditions at 200 °C, 230 °C, and 260 °C (reaction time: 60-120 min, biomass amount 10-20g).

Table S5. ANOVA table for TOC removal (%).

Source	DF	Adj SS	Adj MS	F-Value	P-Value
**Model**	14	71.4994	5.1071	3.61	0.009
**Linear**	4	43.4764	10.8691	7.68	0.001
HC added (g)	1	28.7019	28.7019	20.28	0.000
Catalyst Loading, g/L	1	0.8333	0.8333	0.59	0.455
pH	1	9.5930	9.5930	6.78	0.020
H_2O_2 Concentration, mM	1	4.3483	4.3483	3.07	0.100
**Square**	4	12.6346	3.1587	2.23	0.114
HC added (g)*HC added (g)	1	1.1842	1.1842	0.84	0.375
Catalyst Loading, g/L* Catalyst Loading, g/L	1	1.0218	1.0218	0.72	0.409
pH*pH	1	0.4003	0.4003	0.28	0.603
H_2O_2 Concentration, mM*H_2O_2 Concentration, mM	1	8.4300	8.4300	5.96	0.028
**2-Way Interaction**	6	15.3884	2.5647	1.81	0.164
HC added (g)*Catalyst Loading, g/L	1	2.9593	2.9593	2.09	0.169
HC added (g)*pH	1	7.5294	7.5294	5.32	0.036
HC added (g)* H_2O_2 Concentration, mM	1	0.0000	0.0000	0.00	0.998
Catalyst Loading, g/L*pH	1	3.5835	3.5835	2.53	0.132
Catalyst Loading, g/L*H_2O_2 Concentration, mM	1	1.2885	1.2885	0.91	0.355
pH*H_2O_2 Concentration, mM	1	0.0277	0.0277	0.02	0.891
**Error**	15	21.2328	1.4155		
**Lack-of-Fit**	10	17.7371	1.7737	2.54	0.158

**Pure Error**	5	3.4957	0.6991
**Total**	29	92.7323	

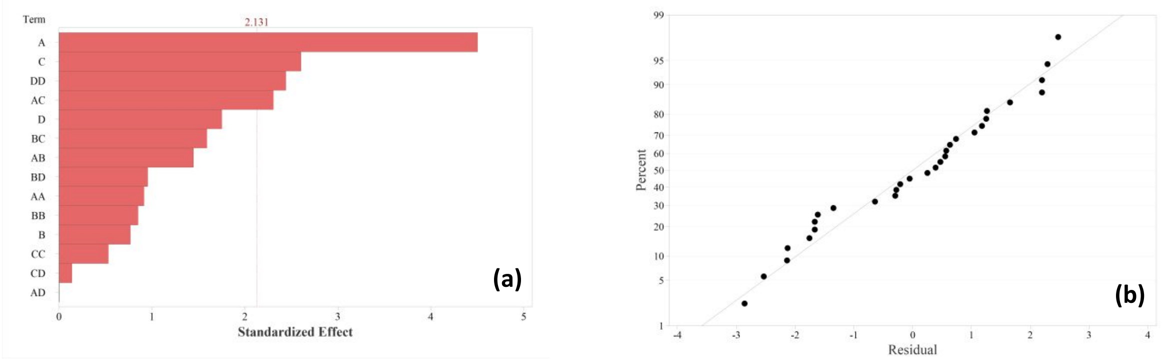

Figure S5. a) Pareto chart of standardized effect (A: HC added (g), B: catalyst loading (g/L), C: pH, and D: H_2O_2 concentration (mM)) and b) normal probability of residuals for TOC removal (%).

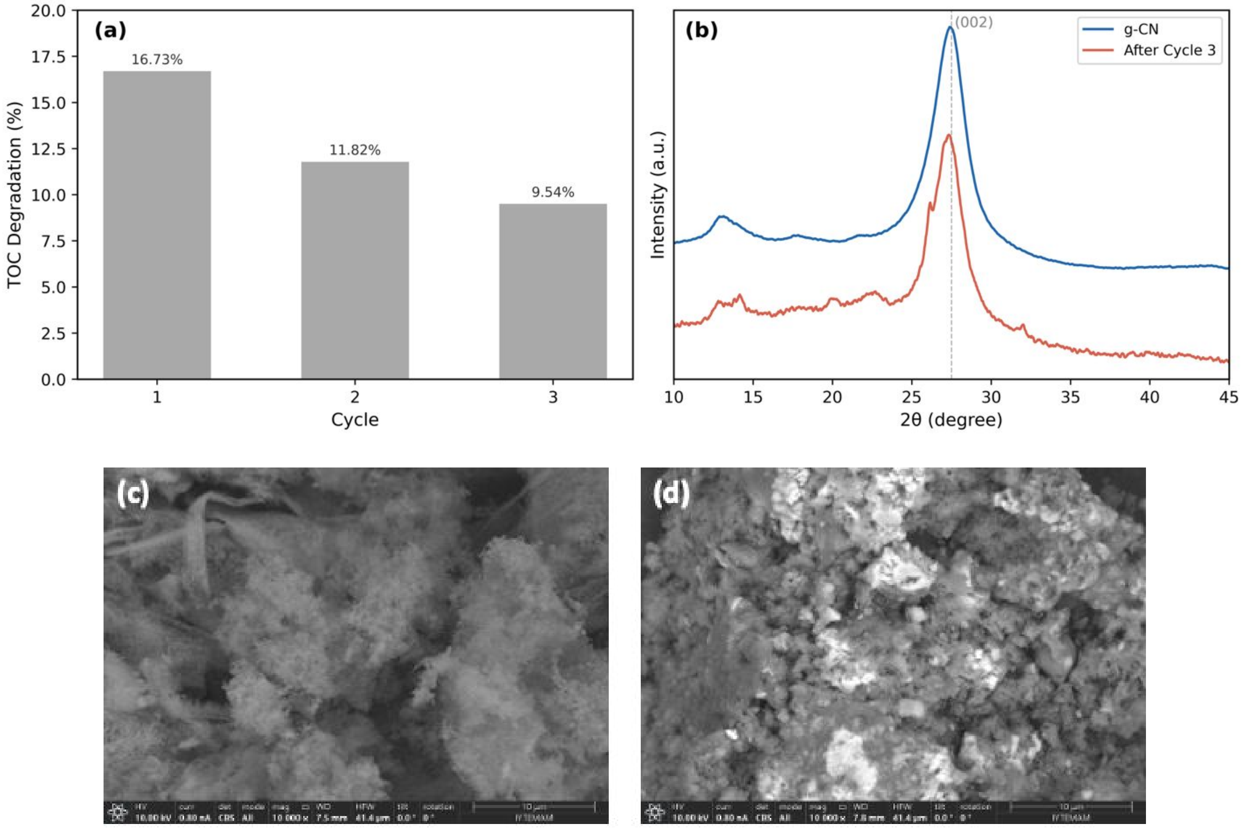

Figure S6. a) TOC degradation efficiency of g-CN over 3 cycles, b) XRD patterns before and after use, and c-d) SEM images of fresh catalyst, and spent catalyst after 3rd cycle
